# Partial embryo loss caused by disruption of the placental hormone CTRP6 coincides with dNK cell abnormalities during pregnancy

**DOI:** 10.1016/j.isci.2025.114434

**Published:** 2025-12-13

**Authors:** Hairui Fan, Xiaolei Chen, Cui Du, Shuai Chen, Wenzhe Yu, Yuchen Tang, Yifu Wang, Haifei Wang, Wenbin Bao, Bin Cao, Ming-an Sun

**Affiliations:** 1 Institute of Comparative Medicine, College of Veterinary Medicine, Yangzhou University, Yangzhou, Jiangsu, China; 2 College of Animal Science and Technology, Yangzhou University, Yangzhou 225009, Jiangsu, China; 3 Fujian Provincial Key Laboratory of Reproductive Health Research, Department of Obstetrics and Gynecology, Women and Children’s Hospital, School of Medicine, Xiamen University, Xiamen 361102, Fujian, China; 4 Joint International Research Laboratory of Important Animal Infectious Diseases and Zoonoses of Jiangsu Higher Education Institutions, Yangzhou, Jiangsu, China; 5 Jiangsu Co-innovation Center for Prevention and Control of Important Animal Infectious Diseases and Zoonosis, Joint International Research Laboratory of Agriculture and Agri-Product Safety of Ministry of Education of China, the Interdisciplinary Center for Zoonotic Diseases and Biosafety, Yangzhou University, Yangzhou, Jiangsu, China

**Keywords:** biological sciences, molecular biology

## Abstract

The proper embryo maintenance depends on tight maternal-fetal communications. C1q/TNF-related protein 6 (*C1qtnf6*)—encoding a hormone-like CTRP6 protein—exhibits placenta-enriched expression and its deficiency causes partial fetal loss. Here, we demonstrate that abundant *C1qtnf6* expression is distinctive for hemochorial placentae, which have deep uterine invasion. *C1qtnf6* is most highly expressed in human extravillous trophoblasts and mouse spiral artery-associated trophoblast giant cells, which are functionally analogous cells that invade uterines. The partial embryo loss is mainly due to impaired CTRP6 production by placenta, and key natural killer effectors in maternal-fetal interface (MFI) are impaired by C1qtnf6-deficiency. C1qtnf6-deficiency simultaneously impairs the amount and effector production of decidual NK (dNK) cells, yet the placental structure and spiral artery remodeling appear normal. CTRP6 injection rescues the dNK cell abnormality and alleviates embryo loss. Overall, the partial embryo loss by CTRP6-deficiency coincides with dNK cell abnormality, which highlights the importance of MFI immune microenvironment for embryo maintenance.

## Highlights

• Abundant *C1qtnf6* expression is distinctive for hemochorial placentae
• Impaired placental-secretion of CTRP6 causes partial embryo lethality
• C1qtnf6-deficiency leads to multifaceted abnormality of dNK cells
• CTRP6 injection restores dNK cell abnormality and alleviates fetal loss

## Graphical abstract

**Figure:**
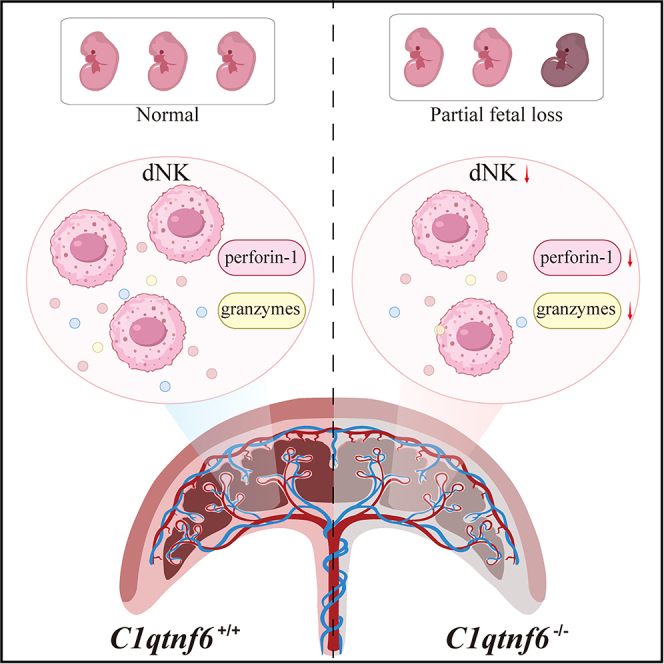

## Introduction

In eutherian mammals, pregnancy is a complicated process involving the tight intercellular communication between the maternal and fetal compartments.^1^ At the fetal side, the placenta not only supports the nutrient/waste exchange between fetus and mother, but also secretes various hormones to sustain the proper embryo maintenance.^2^ Accordingly, placentation defects lead to fetal absorption, growth restriction and gestation disorders, and are identified as the major causes of embryonic lethality in various mutant mice.^1^^,^^3^ At the maternal side, the uterus responses actively to the fetal-side signals and the stromal cells surrounding the implanting embryo are reprogrammed to form the so-called decidua, which supports the growth of the semi-allogenic fetus.^4^^,^^5^ Decidualization is a major feature for the formation of maternal-fetal interface (MFI), and during this process, the spiral arteries undergo remarkably remodeling to facilitate the efficient nutrients/waste exchange between the mother and fetus.^6^^,^^7^

The MFI is composed of the mother-derived decidua and fetus-derived placenta.^5^ In human, the trophoblast cells differentiate into syncytiotrophoblast (STB) and extravillous trophoblast (EVT), with the latter show high invasiveness and are critical for spiral artery remodeling.^6^ Meanwhile, the decidua is abundant with immune cells—such as nature killer (NK) cells, macrophages, T cells, and dendritic cells—with NK cells particularly abundant and extensively studied.^8^^,^^9^^,^^10^ The decidual NK (dNK) cells comprise the majority (∼70%) of decidual immune cells.^5^^,^^11^ Unlike circulating NK cells which attack infected and damaged cells, dNK cells primarily regulate trophoblast invasion, promote immune tolerance, and remodel the spiral arteries.^11^^,^^12^^,^^13^^,^^14^^,^^15^^,^^16^ Interestingly, EVTs express unique MHC molecules, which enable them to act as major ligands for NK cells.^17^^,^^18^ Despite mouse and human both own the highly invasive hemochorial placentae, their placental structure and trophoblast cell types differ remarkably. The mouse placenta is labyrinth shape (but not the villous shape in human) and lacks EVTs, and instead, the spiral artery trophoblast giant cells (SpA-TGCs) play EVT-like roles.^1^ The abnormality of dNK cells is associated with gestation diseases, such as miscarriage, embryo loss, stillbirth, growth restriction, and fetal neuroimmune disorders.^16^^,^^19^^,^^20^^,^^21^^,^^22^^,^^23^^,^^24^ Collectively, the maternal-fetal dialogue is critical for the proper gestation and embryo growth, yet the mechanism remains incompletely understood.

The placenta secretes various hormones and cytokines to regulate gestation. *C1qtnf6* (C1q/TNF-related protein 6) is a gene highly expressed in placenta, which encode a hormone-like CTRP6 protein.^25^ Recently, we compared the transcriptomes across dozens of human tissues and identified *C1qtnf6* (but not any other 14 members of the same gene family) as one of the 339 placenta-enriched genes,^26^ and found it has particularly high expression in EVTs and is bound by core trophoblast transcription factors.^27^ CTRP6 is a glycoprotein bearing both C1q and TNF-like domains, hence is believed to play immune-related functions.^28^ So, does the placenta secrete the CTRP6 protein to fulfill specific roles critical for pregnancy maintenance? Previous GWAS studies linked C1qtnf6 to pre-eclampsia,^29^ thyroid dysfunction during pregnancy,^30^ and multiple autoimmune diseases.^31^^,^^32^ Interestingly, one study found that CTRP6 can be used to treat induced arthritis and noticed that *C1qtnf6*^−/−^ embryos are semi-lethal in mice.^33^ In addition, CTRP6 was reported to inhibit the formation of C3bBb during complement alternative pathway activation in age-related macular degeneration.^34^ Despite of these studies, how the placenta-secreted CTRP6 protein regulate gestation remains unclear.

Here, we demonstrate that the abundant expression of *C1qtnf6* is a unique and conserved feature for the highly invasive hemochorial placentae. Its deficiency leads to partial fetal loss in mice and this process is accompanied by reduced abundance and malfunction of dNK cells. This study provides new insights about how invasive placentae secrete CTRP6 hormone to regulate MFI microenvironment and embryo maintenance.

## Results

### Abundant expression of C1qtnf6 is a distinctive feature for hemochorial placentae

**Figure 1:**
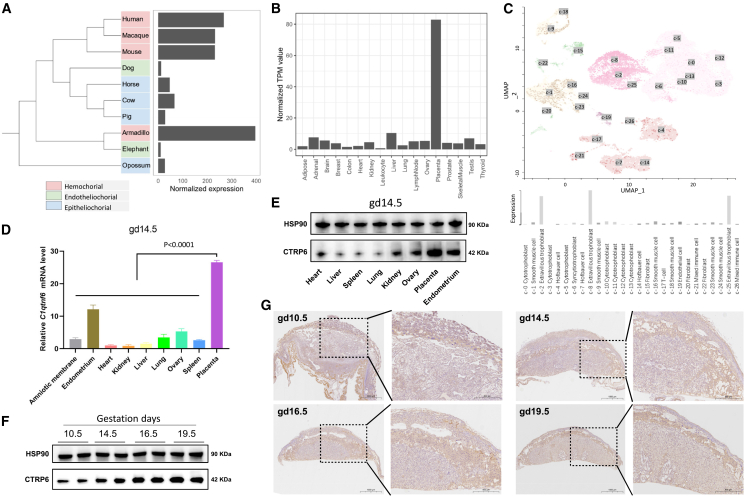
Abundant expression of C1qtnf6 is conserved in hemochorial placentae (A) Comparison of C1qtnf6 expression across different types of placentae in ten mammalian species. The three types of placentae are denoted by colors. The normalized expression levels are calculated for the 1-to-1 orthologous genes across all compared species, with the values for C1qtnf6 visualized as bar plot alongside the species tree. (B) Comparison of C1qtnf6 expression across 17 different human tissues. The bars show the normalized TPM values as calculated from RNA-seq data. (C) Comparison of C1qtnf6 expression across different human trophoblast cell types. The UMAP for scRNA-seq data are adopted from HPA database. The barplot is redrawn based on the data retrieved from HPA, and it visualized the averaged expression of C1qtnf6 in different cell clusters. (D) Comparison of C1qtnf6 expression across different mouse tissues at gd14.5. This result is based on RT-qPCR experiment, with GAPDH as control. *n* = 3. (E) Comparison of the CTRP6 protein abundance in different mouse tissues at gd14.5 by using western blot. (F) Comparison of CTRP6 protein abundance in placenta during different gestation stages. (G) Immunofluorescent imaging of CTRP6 protein in mouse MFI of different gestation stages. Scale bars: 1000 μm (Left) and scale bars: 500 μm (Right). Data are represented as mean ± SD. Statistical analyses were performed by using two-tailed Student’s *t* test.

Previous studies by others and us identified *C1qtnf6* as a gene with placenta-enriched expression in human and mouse,^26^^,^^27^^,^^33^ yet its expression profile in different mammals, trophoblast cells and gestation stages remains obscure. Here, we first compared the placental RNA-seq data of ten mammalian species (Table S1), which cover the three types (hemochorial, endotheliochorial, and epitheliochorial) of placentae. We found that *C1qtnf6* has high expression in all analyzed hemochorial placentae (human, macaque, mouse, and armadillo) that bearing high invasiveness, yet is lowly expressed in the non-invasive epitheliochorial and endotheliochorial placentae (Figure 1A). Transcriptomic comparison across tissues further demonstrates that *C1qtnf6* has placenta-enriched expression in both human and mouse (Figures 1B, S1A, Table S1). In addition, the expression analysis of C1qtnf family genes in mouse placentae by using RT-qPCR showed that *C1qtnf6* has placenta-enriched expression in mouse (Figure S1B). Regarding different trophoblast cells, scRNA-seq data suggests that *C1qtnf6* expression is largely restricted to human EVTs and mouse SpA-TGCs (Figures 1C, S2, Table S1), which are functionally equivalent cell types in primates and rodents. Functionally, EVTs and SpA-TGCs are both highly invasive trophoblast cells that cooperate with dNK cells to regulate spiral artery remodeling in decidua, which is interesting given the remarkable structural differences (villous vs. labyrinth structure) of primate and rodent placentae.^2^^,^^5^^,^^7^ Indeed, recent single-cell studies identified *C1qtnf6* as cell-specific gene for both human EVTs and mouse Spa-TGCs at MFI.^35^^,^^36^ Our comparison of differentiated human trophoblast stem cells (TSCs) also revealed the particularly high expression of *C1qtnf6* in EVTs relative to TSCs and STBs.^27^ Therefore, *C1qtnf6* has high expression in hemochorial placentae, particularly in EVTs and SpA-TGCs which are the most invasive trophoblast cell types for primates and rodents, respectively.

We then focused on mice to inspect the RNA and protein abundance of *C1qtnf6* across tissues and during different gestation stages. We first compared placenta and seven other tissues at gestation day 14.5 (gd14.5) by using RT-qPCR and confirmed the highest expression of *C1qtnf6* in placenta (Figure 1D). Of note, we also noticed the moderate expression of *C1qtnf6* in endometrium, which is the maternal tissue supporting embryo implantation and placental development.^2^ We further inspected the protein level in different mouse tissues by using western blotting, and as expected, CTRP6 protein is most abundant in placenta (Figure 1E). Further comparison of different gestation stages demonstrates the gradually increased levels of CTRP6 protein in placenta as well as serum (Figures 1F and S3). Immunofluorescent imaging confirmed that the distribution of CTRP6 protein is largely restricted to the trophoblastic region of the MFI (Figure 1G)—matching its enriched expression in SpA-TGCs as suggested by scRNA-seq data. Together, the highly abundant expression of *C1qtnf6* appears as a unique feature for hemochorial placentae which bearing high invasiveness, implying the placental-secreted CTRP6 protein may play critical roles in invasive placentae.

### Impaired placental-secretion of CTRP6 hormone causes partial embryo lethality

**Figure 2:**
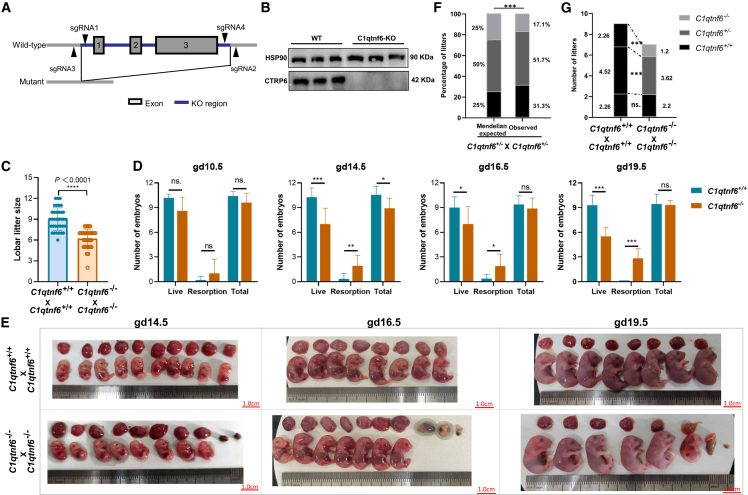
Partial embryo lethality in mice due to impaired placental-secretion of CTRP6 protein (A) Scheme for the CRISPR engineering of the C1qtnf6 genes in mice. The three exons are deleted from C1qtnf6 gene. (B) Western blot confirmed the lack of CTRP6 protein in placenta of homozygous C1qtnf6-KO mice. (C) Comparison of the litter size between WT and homozygous C1qtnf6-KO mice. Statistical test was performed by using two-tailed Student’s *t* test. *n* = 35. (D) Comparison of the embryo resorption numbers between WT and homozygous C1qtnf6-KO mice at different gestation stages. Statistical test was performed by using two-tailed Student’s *t* test. *n* ≥ 5. (E) Visual inspection of the fetal absorption in homozygous C1qtnf6-KO mice at different gestation stages. Scale bars: 1.0 cm. (F) Comparison of the fetus numbers of different genotypes in crossed heterozygous mice relative to Mendelian expectation. Statistical test was performed by Chi-squared test. (G) Comparison of the fetus numbers of different genotypes in crossed heterozygous mice relative to the expected numbers estimated from WT mice. Data are represented as mean ± SD. Statistical analyses were performed by using two-tailed Student’s *t* test. ∗*p* < 0.05, ∗∗*p* < 0.01, ∗∗∗*p* < 0.001.

While the partial fetal loss in *C1qtnf6*-deficient mice was previously noticed by an arthritis-focused study,^33^ the detailed characteristics and underlying mechanism of this phenotype remains unclear. To clarify the role of CTRP6 protein during gestation, we applied CRISPR-Cas9 engineering to generate *C1qtnf6*-knockout (C1qtnf6-KO) mice (Figure 2A). The genotype of the mutant mice was validated by PCR and Sanger sequencing (Figures 2B and S4). The *C1qtnf6*-deficient mice are fertile and look normal even after maturation, so we focused on comparing the embryo phenotype in wild-type (WT) and C1qtnf6-KO mice during gestation. Interestingly, we observed evident sign of fetal resorption as represented by the significantly decreased litter size of 31.0% (9.03 vs. 6.23) in homozygous C1qtnf6-KO mice (Figure 2C, Table S2). Closer inspection of different gestation stages suggests that the trend for fetal resorption appeared at as early as gd10.5, and became statistically significant after gd14.5 (Figures 2D, 2E, and S5; Table S3). Nevertheless, the weights of the retained fetuses and their placentae are comparable (or even higher for early gestation stages) in C1qtnf6-KO relative to WT mice (Figure S6). Interestingly, the variances of fetal and placental weight are remarkably increased in C1qtnf6-KO relative to WT mice for most gestation stages (Figure S6). On the other hand, the general placental structure and spiral artery remodeling appear to be largely unaffected according to HE staining and CD31/α-SMA co-immunostaining (Figure S7).

Despite that CTRP6 protein is predominantly produced by placenta, it is also detectable in endometrium (Figure 1E), which is the maternal tissue supporting placentation. Thus, it is desirable to further clarify whether the observed fetal loss is due to the impaired production of CTRP6 from the fetal (placenta) or maternal (endometrium) side. For this purpose, we performed mating between heterozygous C1qtnf6-KO mice (*C1qtnf6*^+/−^ × *C1qtnf6*^+/−^). Under this setting, the maternal uterine constantly bear the heterozygous genotype, therefore the embryos of different genotypes (*C1qtnf6*^+/+^, *C1qtnf6*^+/−^, *C1qtnf6*^−/−^) can be reasonably compared. As expected, the frequency of *C1qtnf6*^−/−^ offspring is significantly lower than expected by Mendelian ratio (Figure 2F, Table S2). When comparing against the averaged fetus numbers observed in WT mice (2.26:4.52:2.26 by Mendelian ratio), we found that the number of *C1qtnf6*^−/−^ embryos (1.2 vs. 2.26, *p* = 9.2e-6) is significantly lower than expected, *C1qtnf6*^+/−^ embryos (3.62 vs. 4.52, *p* = 0.003) are moderately low than expected numbers, while *C1qtnf6*^+/+^ embryos are similar (2.2 vs. 2.26, *p* = 0.959) to expectation (Figure 2G). Hence, the amount of placenta-secreted CTRP6 protein has predominant role for embryo maintenance. Together, the fetal absorption observed in *C1qtnf6*-deficient mice is mainly due to the impaired CTRP6 secretion from the placenta but not uterine, indicating that the fetal placentae actively secrete CTRP6 to mediate proper embryo maintenance.

### C1qtnf6-deficiency impairs the expression of crucial NK cell genes at maternal-fetal interface

**Figure 3:**
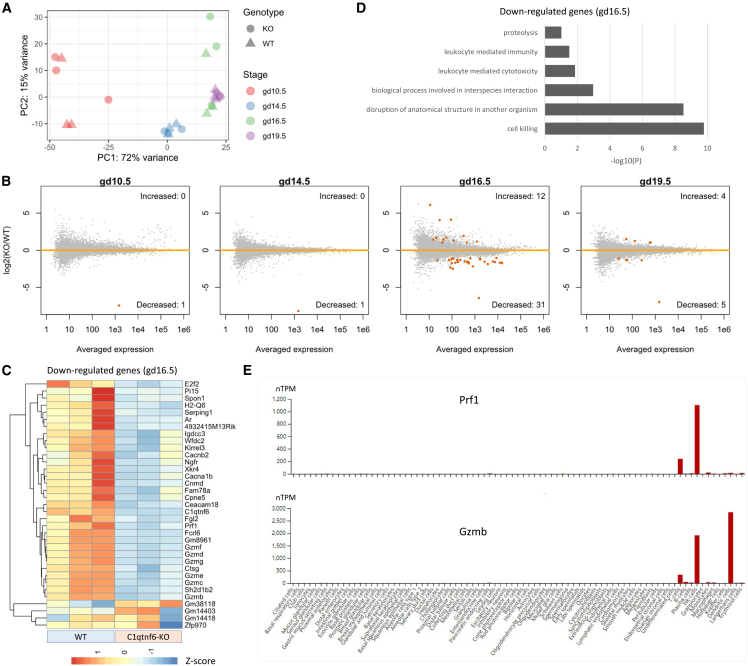
Impaired expression of NK cell genes at MFI after C1qtnf6-deficiency (A) Clustering of different WT and C1qtnf6-KO samples based on PCA analysis. The top 500 genes with the highest variances are used for analysis. (B) MA plots show the differential expression results between WT and C1qtnf6-KO mice. Significant DEGs are highlighted by color. (C) Expression patterns for the 31 genes downregulated in C1qtnf6-KO mice at gd16.5. The expression levels at gd16.5 are visualized. The color gradients show the row *Z* score calculated from normalized read counts. (D) Top enriched GO terms for the 31 genes downregulated in C1qtnf6-KO mice at gd16.5. (E) Data from HPA database show that *Prf1* and *Gzmb* both show NK-cell-enriched expression across different cell types.

Given the abundant expression of *C1qtnf6* in the specialized trophoblast cell types (e.g., human EVTs and mouse SpA-TGCs) that invade uterine, we speculate CTRP6 protein may be secreted to regulate the decidual microenvironment. To test it, we performed transcriptomic comparison between the MFI of WT and homozygous C1qtnf6-KO mice collected at different gestation stages. We observed a highly stage-specific profile regarding the gene expression profiles and alterations after C1qtnf6-KO (Figures 3A and 3B), which is expectable given the dynamic cellular compositions at MFI during gestation.^1^^,^^5^ At the two stages before placenta maturation (gd10.5 and gd14.5), we identified no other genes apart from *C1qtnf6* itself that show significantly altered expression after C1qtnf6-KO (Figure 3B, Table S3). Impressively, at gd16.5 which is a stage shortly after placenta maturation, we identified 43 differentially expressed genes (DEGs) at the MFI of C1qtnf6-KO mice, including 31 downregulated and 12 upregulated genes (Figure 3B, Table S3). On the other hand, we only identified nine DEGs at gd19.5 (Figure 3B, Table S3).

After uncovering the altered gene expression after C1qtnf6-deficiency, we further investigated the affected biological processes. We performed gene ontology (GO) analysis of the 31 downregulated genes at gd16.5, and interestingly, those genes are highly associated with immune related functions, particularly cell killing (Figure 3D). Indeed, many downregulated genes—including perforin (*Prf1*), multiple granzyme genes (*Gzmb/c/d/e/f/g*), and Fc receptor like 6 (*Fcrl6*)—show NK-cell-specific expression according to the data from HPA database (Figures 3E and S8). Importantly, perforin and granzymes are widely regarded as the critical effector molecules for NK cells.^14^^,^^37^ NK cells are also the most abundant and important immune cell types at MFI, with their roles during gestation reported by numerous studies,^9^^,^^13^^,^^16^ including some linking their abnormal production of granzyme to placental defects and pregnancy loss.^10^^,^^38^^,^^39^ Apart from NK-cell-related genes, we also noticed a few other genes with impaired expression. For example, *H2-Q6* is an MHC-1 gene which may promote antigen recognition by NK cells,^40^ androgen receptor (*Ar*) is a reproductive steroid hormone receptor highly expressed in endometrium,^41^ and E2F2 is a transcription activator who’s ablation causes embryo lethality.^42^

Overall, a major finding from the transcriptomic comparison is that C1qtnf6-deficiency leads to the impaired expression of NK-cell-related genes at MFI. Given the close contact between SpA-TGCs and dNK cells and the crucial roles of dNK cells during gestation, we speculate CTRP6 protein may be secreted by SpA-TGCs to regulate dNK cells, hence influence the MFI immune microenvironment.

### Multifaceted abnormality of dNK cells appears at the maternal-fetal interface of C1qtnf6-deficient mice

**Figure 4:**
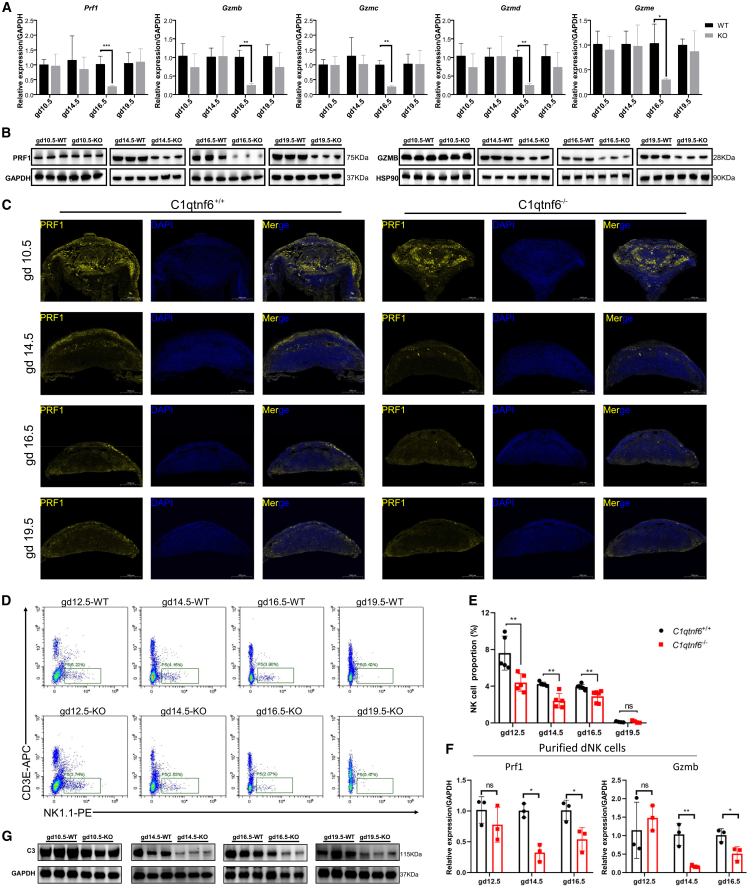
Multifaceted abnormality of dNK cells at the MFI of C1qtnf6-deficient mice (A) RT-qPCR analysis of the altered expression of key NK cell effector genes at the MFI of C1qtnf6-deficient mice. *n* = 3. (B) Similar to A, but examined the protein abundance of PRF1 and GZMB by using western blot. (C) Immunofluorescent imaging of the PRF1 distribution at the MFI of WT and C1qtnf6-deficient mice. Scale bars: 1000 μm. (D) Comparison of the cell amounts of dNK cells at the MFI in WT and C1qtnf6-deficient mice by using FACS sorting. (E) Similar to D, but compared the percentage of sorted dNK cells in different samples. *n* = 4. (F) RT-qPCR analysis of the expression of Prf1 and Gzmb in the dNK cells purified from the MFI of WT and C1qtnf6-deficient mice. *n* = 3. (G) Comparison of the C3 protein abundance at the MFI of WT and C1qtnf6-deficient mice. *n* = 3. Data are represented as mean ± SD. Statistical analyses were performed by using two-tailed Student’s *t* test. ∗*p* < 0.05, ∗∗*p* < 0.01, ∗∗∗*p* < 0.001.

Previous studies suggest that dNK cells regulate spiral artery remodeling and trophoblast invasion, and their absence can cause decreased fetus viability.^5^^,^^7^^,^^11^^,^^21^ Given the impaired expression of NK cell genes after C1qtnf6-deficiency (Figure 3), we next assessed the properties of this specific cell type in MFI samples collected from WT and C1qtnf6-deficient mice at different gestation stages. In line with the RNA-seq data, RT-qPCR validated the stage-specific reduction of *Prf1* and *Gzmb/c/d/e* expression at gd16.5 in C1qtnf6-deficient mice (Figure 4A). We then examined the protein abundance of PRF1 and GZMB by immunoblotting. Interestingly, we found that while their reduction is most evident at gd16.5, this trend in fact appeared as early as gd14.5 and lasted to gd19.5 (Figure 4B). Immunofluorescence imaging of PRF1 protein also confirmed that its reduction occurs as early as gd14.5 in C1qtnf6-deficient mice (Figures 4C and S9). Therefore, the dNK cell abnormality (as reflected by impaired production of key effector proteins) lasts through the middle and late gestation states, which is beyond the gd16.5 when the abnormal expression occurs. Interesting, the partial fetal loss also occurs during these stages (Figure 2), indicating a potential functional link of dNK cell abnormality to the fetal loss in C1qtnf6-deficient mice.

The reduced production of perforin and granzyme can be due to the silencing of these genes, the reduction of dNK cell amounts, or both. To distinguish these possibilities, we used FACS to assess the amount of dNK cell population—marked as CD3E^−^ NK1.1^+^ cells—in the MFI of WT and C1qtnf6-deficient mice (Figure S10). Matching previous studies,^5^^,^^12^ we also observed the gradual decline of dNK cells during gestation (Figure 4D). Interestingly, we observed significantly lower abundance of dNK cells in C1qtnf6-deficienct mice, with this trend appeared as early as gd12.5 and lasted to gd19.5 (Figures 4D and 4E). Of note, we didn’t include gd10.5 for comparison due to the tiny size of the MFI. Hence, the dNK cell population does get decreased in the MFI of C1qtnf6-deficient mice. Even so, does the production of key NK cell effectors in the retained dNK cells remain altered or not? To address it, we collected the dNK cells at different gestation stages by using FACS, and then compared the mRNA level of *Prf1* and *Gzmb* in the purified dNK cells between WT and C1qtnf6-deficient mice. The RT-qPCR results confirmed that both *Prf1* and *Gzmb* show impaired expression in the purified dNK cells of C1qtnf6-deficient mice (Figure 4F). The decrease of dNK cells occurs as early as gd12.5 based on FACS data (Figures 4D and 4E), which is earlier than the altered expression of *Prf1* and *Gzmb* detected at gd16.5 by RNA-seq (Figure 3B), probably because the RNA-seq data are for MFI samples which involve mixed cell populations other than dNK cells. Of note, we noticed reduced levels of C3 at the MFI of C1qtnf6-deficiency mice at as early of gd14.5 (Figure 4G), which may underlie the observed dNK cell abnormality given the response of NK cells to C3 components and the effects of CTRP6 on complement inhibition.^17^^,^^33^^,^^43^ Together, both the dNK cell amounts and the production of key NK cell effectors are impaired in C1qtnf6-deficient mice.

### Injection of CTRP6 protein effectively alleviates the dNK cell abnormality and fetal loss

**Figure 5:**
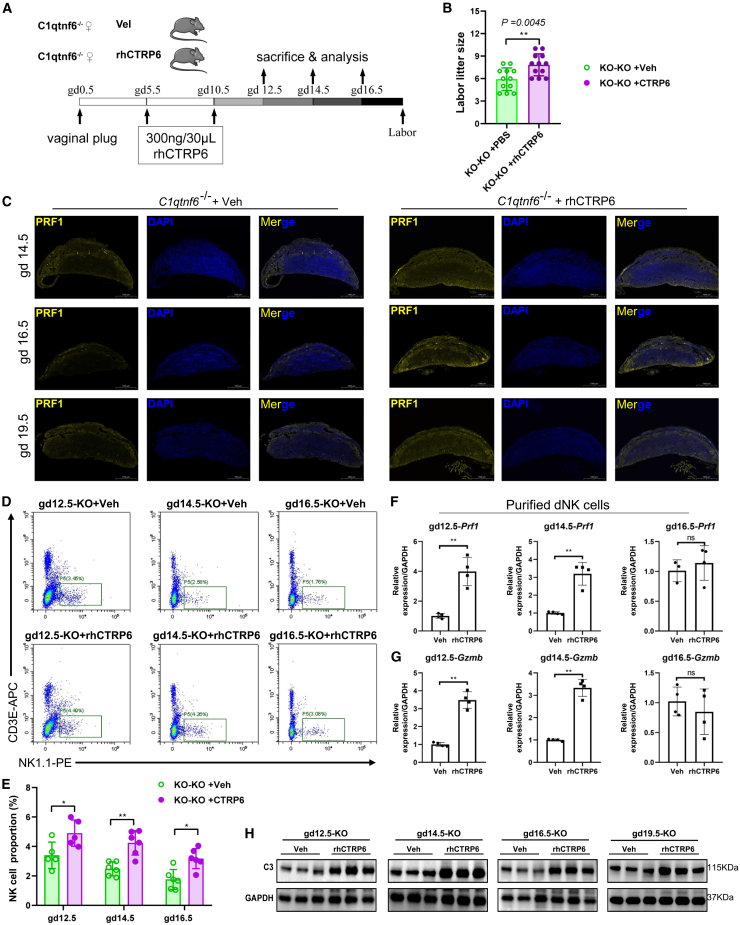
CTRP6 injection alleviates the dNK cell abnormality and fetal loss (A) Scheme figure shows the procedure for intraperitoneal injection of rhCTRP6 protein to pregnant mice. (B) Comparison of the litter sizes in the mice w/wo rhCTRP6 injection. *n* = 12. (C) Immunofluorescent imaging of PRF1 distribution at the MFI in mice w/wo rhCTRP6 injection. Scale bars: 1000 μm. (D) FACS sorting of dNK cells from the MFI of mice w/wo rhCTRP6 injection. (E) Comparison of the dNK cell proportions at the MFI in mice w/wo rhCTRP6 injection. *n* = 5. (F and G) Comparison of the expression of Prf1 and Gzmb in the purified dNK cells from mice w/wo rhCTRP6 injection. *n* = 4. (H) Comparison of the C3 level in the maternal-interface in the mice w/wo rhCTRP6 injection. *n* = 3. Data are represented as mean ± SD. Statistical analyses were performed by using two-sided Student’s *t* test. ∗*p* < 0.05, ∗∗*p* < 0.01.

Given the dNK cell aberrance and partial fetal loss due to C1qtnf6-deficiency, we wonder if such phenotype could be rescued by injection of CTRP6 protein. By re-introducing CTRP6 protein, we can also ascertain the direct influence of MFI by CTRP6 protein. For this purpose, we performed intraperitoneal injection of recombinant human CTRP6 protein (rhCTRP6) in C1qtnf6-deficient mice during gd5.5 and gd10.5 (Figure 5A), and then examined how the embryo and dNK cell phenotypes are changed. Interestingly, the litter size was significantly increased by 24.4% after rhCTRP6 injection (Figure 5B), suggesting the fetal loss is partly rescued. Even though the litter size is still lower than the WT mice (Figure 2E), it is probably because CTRP6 protein has dose-dependent effect.

We further examined the production of key NK cell effectors and found the abundance of PRF1 protein at the MFI is remarkably restored after CTRP6 injection (Figures 5C and S11). To further clarify the effect of CTRP6 protein on dNK cells, we performed FACS sorting to examine the dNK cell population at MFI after CTRP6 injection. Interestingly, the cell proportions of dNK cell populations are remarkably recovered after CTRP6 injection (Figures 5D and 5E). By using the purified dNK cells, we further found that *Prf1* and *Gzmb* both show increased expression after CTRP6 injection at gd12.5 and gd14.5 (Figures 5F and 5G). In line with the decreased C3 level in the MFI of C1qtnf6-KO mice (Figure 4G), we observed remarkably rescued C3 levels after CTRP6 injection (Figure 5H). Further given previous knowledge about the inhibitory role of CTRP6 over the complement alternative pathway,^33^^,^^34^ it indicates the regulatory function of CTRP6 protein over the complement pathway also occurs in the MFI. Together, re-introduction of CTRP6 protein can recuse not only the fetal loss, but also the cell amount and effector production of dNK cells.

## Discussion

The gestation process requires complicated interactions between maternal and fetal sides (decidua vs. placenta), with the crosstalk between dNK cells and invasive trophoblasts (i.e., human EVTs and mouse TGCs) particularly interesting.^14^ The human EVTs and mouse SpA-TGCs can migrate into the uterine tissue, interact with dNK cells which is the most abundant decidual immune cells, to remodel the maternal blood vessels, hence support the growth and development of the conceptus.^7^^,^^44^^,^^45^ Unsurprisingly, the abnormalities of EVTs or dNK cells are both reported to be associated with gestation disorders, such as pre-eclampsia, restricted fetal growth, and recurrent miscarriage.^15^^,^^16^^,^^46^ Trophoblast cells can modulate dNK cell migration, proliferation, and function by secreting a range of cytokines and chemokines.^14^ On the other hand, dNK cells also regulate the differentiation of EVTs and trophoblast invasion in a paracrine manner.^11^^,^^18^ Therefore, the regulation of trophoblast on dNK cells and the decidual immune microenvironment involves a complex network of secreted factors. Our study highlights the function of the hormone-like CTRP6 protein which is secreted by invasive trophoblasts to mediate embryo maintenance, and for the first time shows that the abnormality of dNK cells in MFI is an evident phenotype for C1qtnf6-deficient mice.

While placenta is owned by all eutherian mammals, obvious differences exist across species regarding the shape, structure, cellular composition and molecular feature.^2^^,^^26^ Human and mouse both own the hemochorial placenta, in which maternal blood is in direct contact with trophoblast cells.^44^^,^^47^ To learn more on the links of C1qtnf6 to placenta, we compared different mammals, tissues, trophoblast cells, and gestation stages. Interestingly, transcriptomic analysis showed that abundant C1qtnf6 expression is a unique and conserved feature for hemochorial placentae, which contrasts with all other inspected mammals owning endotheliochorial or epitheliochorial placentae. Specifically, transcriptomic analysis of the MFI and cultured trophoblast cells^27^^,^^35^^,^^36^ uncovered that *C1qtnf6* shows particularly high expression in human EVTs and mouse SpA-TGCs which are functionally analogous cell types that migrate into uterine to facilitate its remodeling. In addition, the expression of *C1qtnf6* is highly specific for placenta and its protein abundance is gradually increased during gestation. This is in contrast to its paralogous members, such as *C1qtnf1* showing ubiquitous expression and other members that are absent from placenta.^25^ These evidences highlight that *C1qtnf6* is a gene obtained specialized roles in invasive placenta during mammalian evolution, and its specific expression in EVTs and SpA-TGCs imply it may be secreted to regulate the maternal decidua.

We inspected the roles and CTRP6 protein during gestation by generating C1qtnf6-KO mice, focusing on its influence at the MFI. Direct comparison between homozygous wild-type and mutant mice confirmed the partial fetal loss (by ∼45%) in mutant mice, and the embryo absorption occurs after gd14.5 when the placenta gets matured. Crossbreeding of heterozygous mice revealed that the fetal loss is due to the impaired production of CTRP6 by the placenta rather the uterine, since only homozygous mutant fetus have lower than expected numbers. Therefore, we speculate that the homochorial placentae secrete CTRP6 to regulate embryo maintenance. However, while the mice crossing data suggest the importance of placenta-secreted CTRP6 protein for embryo maintenance, the evidence is relatively indirect. Alternatively, embryo transfer experiments of WT vs. C1qtnf6-KO fetus to wild-type recipient female mice would provide further evidence about the role of placenta-secreted CTRP6 protein.

Our transcriptomic analysis hints the abnormality of dNK cells after C1qtnf6-KO, since the downregulation of key NK cell effectors (particularly *Prf1* and multiple *Gzm* genes) is the most evident abnormality at the MFI. This finding is attractive given the close contacts between SpA-TGCs and dNK cells and the pivotal roles of dNK cells for embryo maintenance,^8^^,^^14^ hence we paid special attention on dNK cells. We found the abnormality of dNK cells in C1qtnf6-KO mice is evident: (1) according to FACS data, the amount of dNK cells at MFI is decreased; (2) regarding the purified dNK cells, their production of perforin granzymes is also remarkably decreased. Therefore, CTRP6 protein regulates not only the dNK cell accumulation at decidua but also the malfunction (as reflected by the low production of key effectors) of dNK cells. These data suggests that the abnormality of dNK cells coincides with the partial fetal loss in C1qtnf6-KO mice. Of note, the abnormality of dNK cells starts earlier than gd12.5, which is before the fetal loss appears. This is in line with previous observation that pregnancy disorders occur late yet begin at early stages.^1^ Further given the pivotal role of dNK cells and that both the dNK cell and embryo abnormality can be rescued after CTRP6 inject, it is attractive to speculate a potential causal link between CTRP6-dNK cell axis and embryo maintenance. Nevertheless, we cannot preclude the possibility that other minority cell populations (e.g., T lymphocytes and macrophages) at the MFI may also be disturbed after C1qtnf6-deficiency and contribute to the observed partial fetal loss. Single-cell sequencing experiments and dNK cell depletion or inhibition experiments may further resolve the contribution of dNK cell dysfunction to the fetal loss phenotype after C1qtnf6-deficiency. Despite of this limitation, we suppose that the identified abnormality of dNK cells can at least be regarded as a hallmark for the disrupted immune microenvironment at the MFI as caused by C1qtnf6-deficiency.

Regarding why dNK cells get aberrant after C1qtnf6-KO, multiple evidences suggest it may involve the complement activation. A previous study identified CTRP6 protein as a complement regulator and showed that it can be used to treat induced arthritis by suppressing the alternative complement pathway.^33^ In another study, CTRP6 was reported to inhibit the formation of C3bBb during complement alternative pathway activation in age-related macular degeneration.^34^ Despite that the C3 expression in MFI remained constant (or even increased) after C1qtnf6-KO, we found its protein abundance is decreased—a sign for complement activation and assumption according to previous studies on autoimmune diseases.^17^ Indeed, multiple previous studies linked aberrant complement activation to pregnancy disorders.^48^^,^^49^ For example, an early study found that complement activation is associated with the placental and fetal injury during recurrent miscarriages and intrauterine growth restriction.^50^ Accordingly, several factors for inhibiting complement activation at MFI have also been reported previously, such as the rodent-specific *Crry* which is a complement regulator whose deficiency causes embryonic lethality.^51^ The exact regulatory mechanism of CTRP6 proteins over the complement pathway remain to be further investigated. In practical, CTRP6 may also have the potential to be used for the prevention of embryo loss during gestation.^52^

As the most abundant and critical immune cells in decidua, dNK cells are extensively studied and multiple proteins serving as dNK cell regulators have been reported previously. For example, a key leukocyte signaling molecule named p110δ was reported to regulate dNK cells and macrophages in decidua, hence influence fetus maintenance and growth in mice.^46^ The deficiency of the maternal-secreted BMPR2 protein also causes dNK cell abnormality (regarding both the cell amounts and the expression of *Prf1*) and fetal loss in mice,^53^ resembling the phenotype after C1qtnf6-KO. Recently, it was found that dNK cells can also respond to the cytokine IL-21 (likely produced by macrophages and T cells) during decidualization, thus get enhanced functionality for spiral artery remodeling.^12^ Despite of these studies, the CTRP6 proteins still show several special properties. Compared with these proteins, C1qtnf6 is special in that it shows conserved high expression in hemochorial placentae (unlike the rodent-specific *Crry*) and it is specifically secreted by invasive trophoblasts (but not by maternal cells as for IL-21 and BMPR2) to regulate decidua, thus represents an active strategy to regulate the maternal immune microenvironment. Meanwhile, plenty studies also uncovered the regulation of trophoblasts by dNK cells,^9^^,^^54^ together highlight the complicated cross-talk between the dNK cells and trophoblasts at MFI.

### Limitations of the study

Several limitations of this study should be noted. First, while the multifaceted abnormality of dNK cells is uncovered as the most evident phenomenon after C1qtnf6-deficiency, it remains unclear if dNK cell abnormality plays dominant roles for the observed partial fetal loss. Further study based on dNK cell depletion or inhibition experiments on animal models can provide additional evidence on the contribution of dNK cells for proper embryo maintenance. Second, it remains unclear why the cell amount and effector molecule production of dNK cells can be remarkably affected after C1qtnf6-deficiency. We speculate that instead of direct regulation on dNK cells, it is more possible that regulation of dNK cells by CTRP6 proteins is mediated by the complement pathway—yet additional experiments (such as by using purified dNK cells) are necessary to clarify the underlying regulatory mechanism. Last, apart from the abnormality of dNK cells, it is possible that other immune cell types—such as T cells and macrophages—may also be affected after C1qtnf6-deficiency and contribute to the observed partial embryo loss. We expect that single-cell resolution comparison of the MFI samples between WT and C1qtnf6-KO mice by using scRNA-seq could further characterize the subtle (yet potentially critical) alterations of these minor immune cell populations due to C1qtnf6-deficiency.

## Resource availability

### Lead contact

Further information and requests for resources and reagents should be directed to and will be fulfilled by the lead contact, Ming-an Sun (mingansun@yzu.edu.cn).

### Materials availability

This study did not generate new unique reagents.

### Data and code availability

• Sequence data that support the findings of this study have been deposited in the Gene Expression Omnibus (GEO) database (https://www.ncbi.nlm.nih.gov/geo/) under accession GSE285241.
• The raw experimental data and images have been deposited to FigShare at: https://figshare.com/s/dc82a989f61eb7930798.
• The custom scripts have been deposited to GitHub at: https://github.com/mingansun/CTRP6-project.

## Acknowledgments

We are grateful to all the staffs involved in the mouse care and feeding, and Dr. Binqing Fu for insightful discussions. This research was supported by the grants from the 10.13039/501100001809National Natural Science Foundation of China (32270584 to M.-a.S.), Jiangsu Basic Science (Natural Science) Research Project of Higher Education Institutions (24KJD230003 to H.F.), the Interdisciplinary Center for Zoonoses and Biosecurity of 10.13039/501100007062Yangzhou University, the 111 Project D18007, and the Priority Academic Program Development of Jiangsu Higher Education Institutions (10.13039/501100012246PAPD). This study utilized the computational resource of the Yangzhou University College of Veterinary Medicine High-Performance Computing Cluster.

## Author contributions

M.-a.S. and H.F. conceived and designed the project. M.-a.S., W.B., and B.C. supervised this study. H.F., X.C., W.Y., and H.W. performed the experiments. C.D., M.-a.S., and S.C. performed bioinformatics analysis. Y.T., Y.W., and X.C. were responsible for the feeding of the mice. H.F. and M.-a.S. wrote the manuscript. H.W. and B.C. gave advice and revised the manuscript. All authors have reviewed and approved the paper.

## Declaration of interests

The authors declare no competing interests.

## STAR★Methods

### Key resources table

**Table:** 

REAGENT or RESOURCE	SOURCE	IDENTIFIER
**Critical commercial assays**		

DNA extraction kit	Vazyme	CAT# DC301-01
RNAs using HiScript® Reverse Transcriptase kit	Vazyme	CAT# R201-01
Enzyme-linked immunosorbent assay (ELISA) kit	CUSABIO	CAT# CSB-EL003651HU
BCA protein assay kit	Solarbio	CAT# PC0020
RNAiso	Takara	CAT# 9109
SYBR Green master mix	Vazyme	CAT# Q421

**Antibodies**		

Mouse monoclonal anti- Prf1	Santa Cruz	CAT# sc-374346; RRID:AB_10988266
Mouse monoclonal anti-Gzmb	R&D systems	CAT# Af1865; RRID:AB_2294988
Rabbit monoclonal anti-C3	Abcam	CAT# ab181147
Mouse monoclonal anti-GAPDH	Proteintech	CAT# 10494-1-AP; AB_2263076
Mouse monoclonal anti-HSP90	Proteintech	CAT# 60318-1-lg; RRID:AB_2881429
Rabbit monoclonal anti-CTRP6	Abcam	CAT# ab36900; RRID:AB_731490
anti-mouse CD16/32	BioLegend	CAT# 156603; RRID:AB_2783137
anti-mouse NK1.1	Biogems	CAT# 83712-60
anti-mouse CD3E	Biogems	CAT# 05122-80

**Biological samples**		

placenta	This paper	N/A

**Chemicals, peptides, and recombinant proteins**		

Fetal Bovine Serum	Thermo Fisher Scientific	CAT# 10437-028
DNase Ⅰ	STEMCELL	CAT# 07900
10 × ACK Lysis Buffer	Elabscience	CAT# E-CK-A105
Collagenase Ⅳ	STEMCELL	CAT# 07909
Bovine Serum Albumin	Sigma-Aldrich	CAT# A8806
RhCTRP6	Prospec	CAT# pro-655
DAPI	Invitrogen	CAT# 62248

**Deposited data**		

Single-cell RNA sequencing data	Vento-Tormo et al.^55^	E-MTAB-6701
Single-cell RNA sequencing data	Jiang et al.^35^	GSE59992
RNA-seq data of cow placenta	Armstrong et al.^56^	GSE79121
RNA-seq data of cow placenta	Moradi et al.^57^	GSE194033
RNA-seq data of dog placenta	Armstrong et al.^56^	GSE79121
RNA-seq data of armadillo placenta	Armstrong et al.^56^	GSE79121
RNA-seq data of horse placenta	Coleman et al.^58^	GSE46859
RNA-seq data of horse placenta	Wang et al.^59^	GSE30243
RNA-seq data of human placenta	Necsulea et al.^60^	GSE43520
RNA-seq data of human placenta	Dunn-Fletcher et al.^61^	GSE118285
RNA-seq data of human placenta	Pavličev et al.^62^	GSE87708
RNA-seq data of elephant placenta	Armstrong et al.^56^	GSE79121
RNA-seq data of rhesus monkey placenta	Sun et al.^63^	GSE153082
RNA-seq data of rhesus monkey placenta	Dunn-Fletcher et al.^61^	GSE118284
RNA-seq data of opossum placenta	Armstrong et al.^56^	GSE79121
RNA-seq data of opossum placenta	Necsulea et al.^60^	GSE43520
RNA-seq data of mouse placenta	Dunn-Fletcher et al.^61^	GSE43520
RNA-seq data of mouse placenta	Necsulea et al.^60^	GSE118283
RNA-seq data of pig placenta	Wang et al.^64^	GSE110414
RNA-seq data of mouse placenta	This paper	GSE285241

**Software and algorithms**		

STAR v2.7.3	Dobin et al.^65^	https://github.com/alexdobin/STAR
subread v2.0.0	Liao et al.^66^	https://github.com/ShiLab-Bioinformatics/subread
DESeq2 v1.30.1	Love et al.^67^	https://bioconductor.org/packages/DESeq2
RSEM v1.3.2	Li et al.^68^	https://github.com/deweylab/RSEM
ExprX package v0.0.3	Sun et al.^26^	https://github.com/mingansun/ExprX
Seurat v3.1.5	Stuart et al.^69^	https://satijalab.org/seurat
DAVID	Huang et al.^70^	https://davidbioinformatics.nih.gov
R	Team, R.C^71^	https://www.r-project.org
GraphPad Prism	GraphPad	http://www.graphpad.com

### Experimental model and study participant details

#### Animals

All animal research were approved by the Ethics Committee of Yangzhou University Health Science Center and were conducted according to the institutional ethical guidelines for animal experiments and safety guidelines for gene manipulation experiments (license approval number: SYXK (Su) 2022-0044). The animals involved in this study were all on C57BL/6 background. C57BL/6 wildtype (WT) mice and C1qtnf6-KO (S-KO-14014, strain C57BL/6JCya) mice were obtained from Cyagen Biosciences (Suzhou, China). Mice were bred and maintained in a specific-pathogen-free facility with a 12 h light/12 h dark cycle, ambient temperature of 20°C–24°C, humidity of 30–70% and allowed free access to food and water. To prepare pregnant female mice of different genotypes, we randomly allocated one male and one female mice into each cage. Mating experiment was started at 4:30–5:30 p.m., and then vaginal plug was checked at 8:30–9:30 a.m. in next morning; the existence of vaginal plug was considered as 0.5 days postcoitum (dpc), and was considered as 0.5 days of gestation if pregnancy was confirmed later. The gRNA sequences used for CRISPR/Cas9 engineering are listed in Table S4.

### Method details

#### Mice genotyping

Genotyping of mice was performed by using the standard genomic DNA extracted from toe samples, followed by PCR and gel electrophoresis. The PCR primers for genotyping are listed in Table S5.

#### Titration of CTRP6

Mice were killed under ether anesthesia, and their serum was collected and diluted 10 times with PBS (PH7.4). The CTRP6 level of serum supernatant at different gestation days (No-pregnancy, gd6.5, gd10.5, gd14.5, gd16.5 and gd19.5) was measured using the Enzyme-linked immunosorbent assay (ELISA) kit (CUSABIO, CSB-EL003651HU, Wuhan, China). Each sample was analyzed in triplicate and the mean value was measured.

#### Immunohistochemistry

Placenta samples were fixed in 4% paraformaldehyde overnight. After dehydration, the samples were embedded in paraffin and sectioned at a thickness of 5 μm. Sections were deparaffinized in xylene three times (5 min each), rehydrated in anhydrous ethanol and 95% ethanol twice (5 min each). For H&E staining, the section stained with hematoxylin and eosin. For IHC staining, the sections were incubated in blocking buffer (5% normal donkey serum, 1% BSA, 0.3% Triton X-100 in PBS) for 1 h at room temperature (RT), and then stained with anti-CTRP6 (1:25, Abcam, ab36900), anti-Prf1 (1:25, Santa Cruz, sc-374346) antibodies in blocking buffer at 4°C overnight. After washed 3 times in PBS, the tissue sections were stained with biotin-labeled secondary antibodies, and counterstained with DAPI (62248, Invitrogen, USA) for 5 min. The slides were counterstained with H&E and imaged using an 80i microscope equipped with a camera (Nikon, Tokyo, Japan).

#### Real-time quantitative PCR

The total RNA of placentae was extracted using RNAiso (9109, Takara, Dalian, China), cDNAs were synthesized from the purified RNAs using HiScript Reverse Transcriptase kit (R201-01, Vazyme, Nanjing, China). Quantitative PCR was performed using SYBR Green master mix (Q421, Vazyme, Nanjing, China) in an ABI StepONEPlus Real-Time PCR System (Applied Biosystems, Foster City, CA, USA). The mouse *GAPDH* was selected as an internal control. Each gene was performed in triplicate or more and the relative quantitative of gene expressive was calculated using 2^-ΔΔCt^ method. All the primers used for qRT-PCR were listed in Table S6.

#### Western blotting

The samples were lysed with RIPA buffer supplemented with protease inhibitors for 15 min on ice, scraped and centrifuged at 12,000 rpm at 4 °C for 10 min. After quantified using BCA protein assay kit (PC0020, Solarbio, Beijing, China), equal amounts (20 μg) of denatured proteins were loaded on 10–12% SDS-PAGE gel for electrophoresis, transferred to PVDF membranes (Millipore Corp., Bedford, MA, USA), and then blocked with 5% skim milk (BD, Franklin Lakes, NJ, USA) at room temperature for 1 h. After probing with primary antibodies (Table S6) overnight at 4°C, the membranes washed with TBST (T1081, Solarbio, Beijing, China) and incubated with secondary antibodies (Jackson ImmunoResearch Laboratories, West Grove, PA, USA) at RT for 1 h. Protein bands were visualized using Luminol/Enhancer Reagent (New Cell & Molecular Biotech, Suzhou, China) and then exposed with FluorChem FC3 system (Protein-Simple, CA, United States). Finally, the relative integrated density of each band was digitized with FluorChem FC3 system. GAPDH (Proteintech, 10494-1-AP) or HSP90 (Proteintech, 60318-1-lg) were used as internal control. The primary antibodies and dilution factors are listed in Table S7.

#### RNA-seq analysis

Total RNA for placentae of mice were extracted using Trizol reagent (9109, Takara, Dalian, China) with on-column DNase digestion, and then submitted for library construction by TruSeq stranded mRNA sample preparation kit (Illumina). RNA-seq libraries were sequenced as 150 bp paired-end reads with HiSeq2500 (Illumina) platform. Raw reads were trimmed with Trim Galore v0.6.4, and then aligned to the mouse reference genome (GRCm38) using STAR v2.7.3^65^ with default settings, and then obtained gene-level read counts using the *featureCount* function from subread v2.0.0^66^ with default settings. At last, differentially expressed genes were identified using DESeq2 v1.30.1^67^ with the cutoff: FDR<0.05 and |log2Foldchange|>1. Of note, DESeq2 determines FDR values by using Benjamini-Hochberg approach.

#### Interspecies gene expression comparison

Placental RNA-seq data for ten different mammalian species are collected from multiple studies, as summarized in Table S1. Raw reads were trimmed with Trim Galore v0.6.4. Transcript Per Million (TPM) value for each gene in each sample was calculated with RSEM v1.3.2^68^ with default settings. By using the ExprX package v0.0.3 we previously developed,^26^ we determined the one-to-one orthologues across all compared species and then calculated their normalized TPM values by using TMM approach. The normalized expression levels were subjected to interspecies comparison. The reference genomes used include human (GRCh38), rhesus monkey (Mmul_10), mouse (GRCm38), dog (ROS_Cfam_1.0), horse (EquCab3.0), cow (ARS-UCD1.3), pig (Sscrofa11.1), armadillo (Dasnov3.0), elephant (loxAfr3), and opossum (ASM229v1).

#### scRNA-seq

The scRNA-seq of human and mouse MFI samples are generated by previous studies^35^^,^^36^ and summarized in Table S1. For human scRNA-seq data, we retrieved the processed data of the original study^36^ directly from HPA database,^72^ which include the clustering figures and the averaged gene expression level for each cell cluster. For mouse scRNA-seq, we visualized the data by using Seurat v3.1.5^69^ according to the pipeline shared by the original study,^35^ with the technical details and scripts already made available by the original study.

#### GO enrichment analysis

GO enrichment analyses for differentially expressed genes were performed using DAVID bioinformatics resources.^70^ Only the GO terms from “Biological processes” category were used for visualization.

#### Tissue digestion and cell preparation

For placentae digestion, the tissues were harvested from mice at gd12.5, gd14.5, gd16.5 and gd19.5. Then, the tissues were opened longitudinally, cut into small pieces (∼5 mm^2^), and digested with 1 mg/mL Collagenase Ⅳ (07909, STEMCELL, shanghai, China) and 0.5 mg/mL DNase Ⅰ (07900, STEMCELL, shanghai, China) at 37°C for 30 min. The digested tissues were strained through 70 μm filters, erythrocytes were lysed for 10 min at room temperature (Elsbscience, E-CK-A105), washed with PBS containing 2% FBS, and then count 10^6^ cell/100 μL, followed by antibody staining.

#### Flow cytometry

Digested placenta cells were resuspended in PBS with 2% FBS. Before surface stating, the anti-CD16/32 antibody (BioLegend, 156603) was used to block the Fc receptors. Afterward, the cells were incubated with anti-mouse CD3E (Biogems, 05122-80) and anti-mouse NK1.1 (Biogems, 83712-60) to surface molecules for 30 min at 4 °C, and then washed and resuspended in PBS with 2% FBS.

Flow cytometric analysis was performed on LSRFortessa (BD Biosciences). Data were analyzed with FlowJo software (BD Biosciences). Cell sorting was performed on Aria III cytometers (BD Biosciences) at high purity. The dNK cells from MFI were sorted by the gating strategy of live CD3E^−^ NK1.1^+^.

#### Re-introduction of CTRP6 protein

To examine the therapeutic effect of CTRP6 on dNK cell abnormality and fetal loss, we injected rhCTRP6 (pro-655, Prospec, Israel) to C1qtnf6-deficient pregnancy mice. Virgin female mice at 8 to 10 weeks of age were cohabited with adult males (>12 weeks of age). Vaginal plugs was observed at 5 a.m. the next day, if observed vaginal plugs are considered pregnancy, and recorded as gestation day 0.5. Pregnant mice were gently divided into two groups at random. The experimental group received injections of 300ng/30 μL on gd5.5 and 10.5, while the control group was administered with 30 μL of PBS on the same days Twelve female mice in each of two groups were used to record the litter size. The remaining mice were killed on gd12.5, 14.5, 16.5 and 19.5, and their placenta tissues were collected for further analysis. The content of dNK cells in the placenta was assessed using flow cytometry, and these cells were then analyzed for related gene expression levels.

#### Statistical analysis and visualization

All values are presented as the mean ± SD of at least three independent experiments. Comparisons of two means were analyzed by two-sided Student’s *t* test. *p* < 0.05 was considered as statistically significant, ∗*p* < 0.05, ∗∗*p* < 0.01, ∗∗∗*p* < 0.001. The statistical analyses of Mendelian ratios of C1qtnf6-KO mice were performed by χ2 test. The mice were randomly divided into different experimental groups or divided by their genotyping and gestational days. Most statistical analyses were performed with R statistical programming language^71^ and Prism 8 software (GraphPad Software, La Jolla, CA, USA).

### Quantification and statistical analysis

The qRT-PCR data were analyzed using the 2^ΔΔ^Ct method, and the gene expression levels were presented in a bar chart format. The differential expression analysis of the log2 standardized and scaled data was performed using the FindMarkers function, and the Wilcoxon rank-sum test was applied. All other parameters were set to default values. The data for comparisons between groups or columns were analyzed using GraphPad Prism. The results were presented in the form of mean ± SD, with at least three independent experiments. The “n” numbers in all experiments indicated the number of independent repetitions. The comparison between the two groups was analyzed using the two-tailed Student’s *t* test. A *p*-value of <0.05 was considered statistically significant.
