# Supplementary material for: Partial embryo loss caused by disruption of the placental hormone CTRP6 coincides with dNK cell abnormalities during pregnancy
Source: iScience. 2025 Dec 13;29(1):114434. doi: 10.1016/j.isci.2025.114434 (PMC12818072; doi:10.1016/j.isci.2025.114434)
Supplement: Document S1. Figures S1–S11 and Tables S1–S7 [file mmc1.pdf]

iScience, Volume *29*

Supplemental information

Partial embryo loss caused by disruption of the placental hormone CTRP6 coincides with dNK cell abnormalities during pregnancy

Hairui Fan, Xiaolei Chen, Cui Du, Shuai Chen, Wenzhe Yu, Yuchen Tang, Yifu Wang, Haifei Wang, Wenbin Bao, Bin Cao, and Ming-an Sun

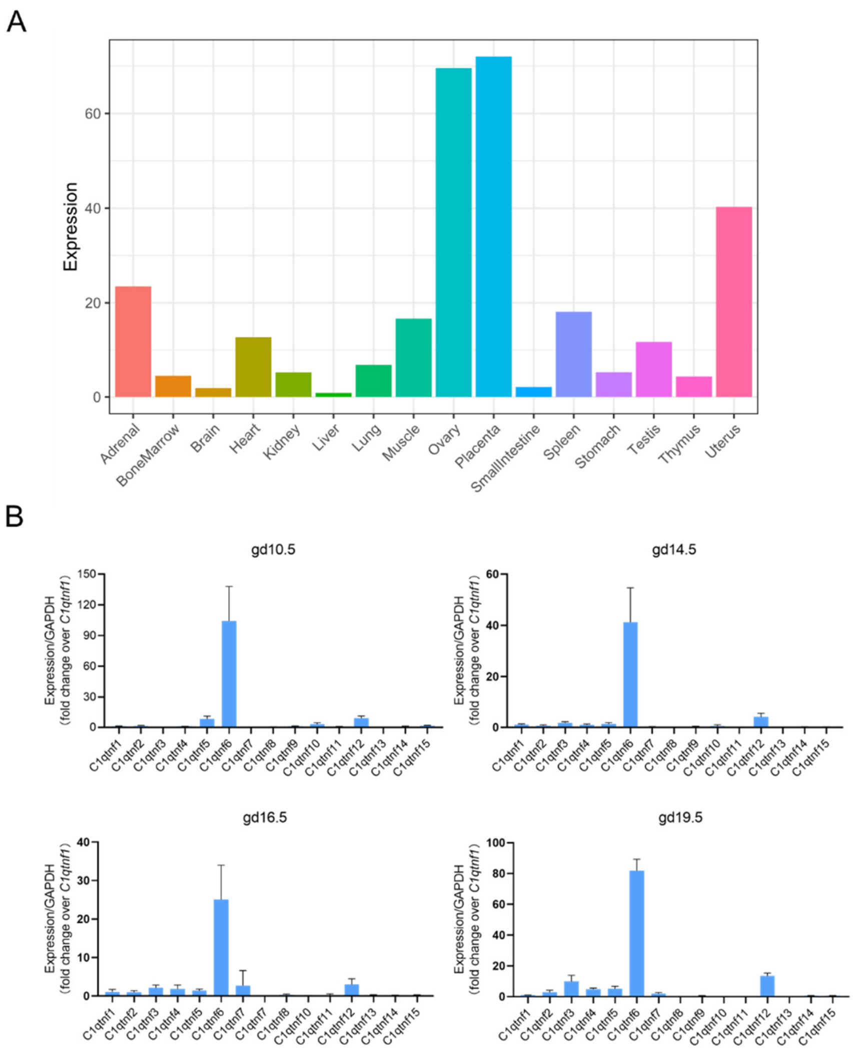

Figure S1. Expression profile of C1qtnf6 across different mouse tissues and the relative expression of C1qtnf family in mouse placentae

(A) The expression of C1qtnf6 in different mouse tissues as measured by normalized TPM values. All the involved data are summarized in Table S1. (B) The relative expression of C1qtnf family mRNA in placentae were determined by qRT-PCR (n = 3).

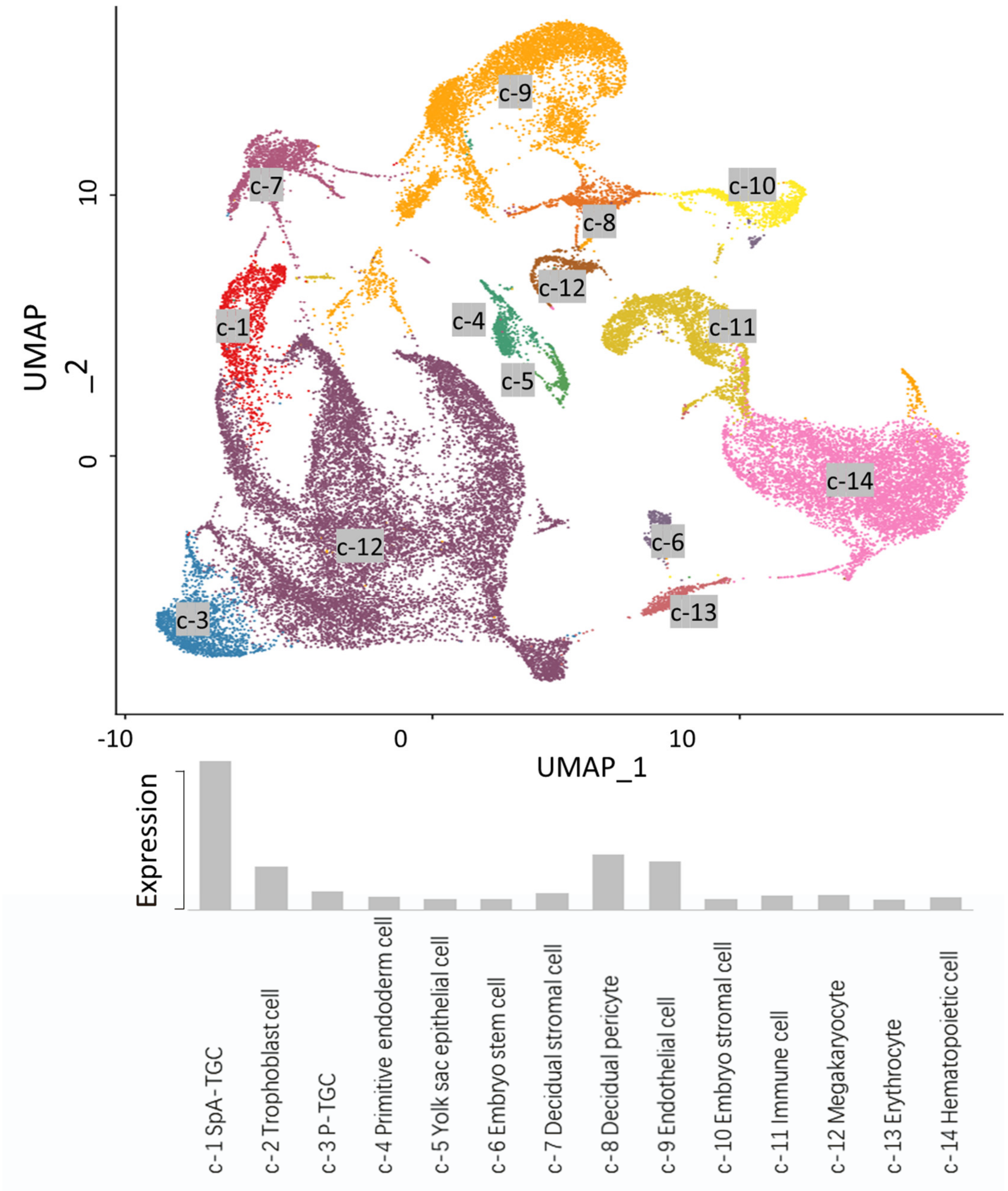

Figure S2. The expression profile of C1qtnf6 in different mouse trophoblast cell types

This figure demonstrates the expression profile of C1qtnf6 in different trophoblast cell types, according to the scRNA-seq data of mouse MFI. The involved data are summarized in Table S1.

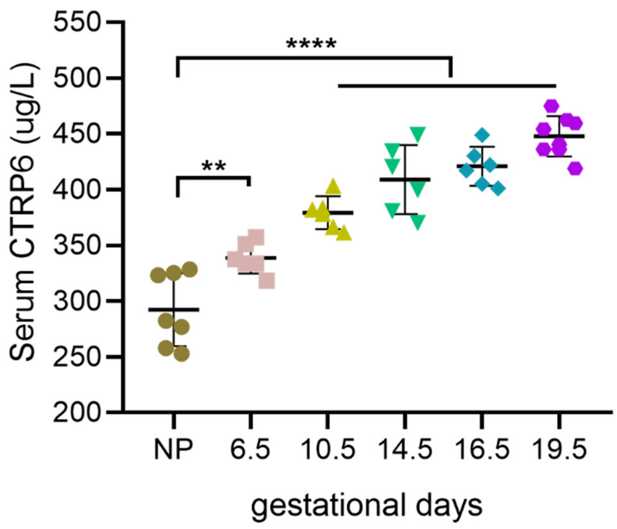

Figure S3. The abundance of serum CTRP6 protein during gestation

This figure demonstrates the gradually increased serum abundance of CTRP6 protein during gestation in mice.

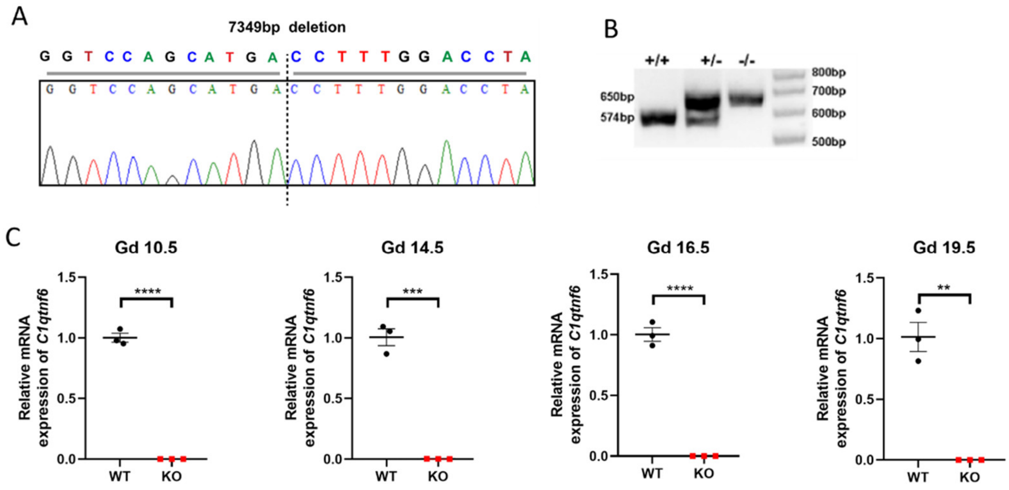

Figure S4. Validation of the CRISPR engineering of C1qtnf6 in mice

(A) Sanger sequencing validated the deletion of 7,344 bp region in C1qtnf6-KO mice. (B) PCR validation of the CRISPR-mediated deletion in heterozygous and homozygous C1qtnf6-KO mice. (C) qRT-PCR validation of decreased levels of C1qtnf6 RNA in C1qtnf6-KO mice.

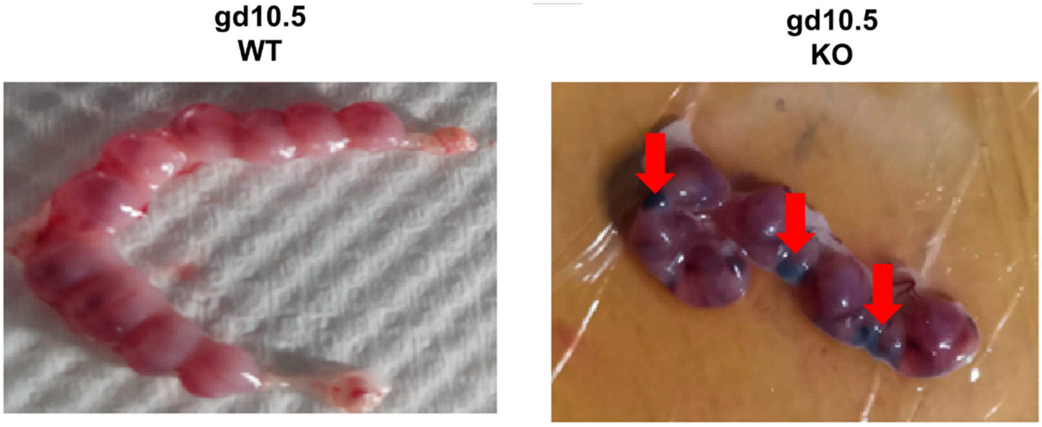

Figure S5. Demonstration of fetal absorption at early gestation stages
This figure demonstrates the fetal absorption at as early as gd10.5 in C1qtnf6-KO mice. This figure is related to Figure 2. Of not, even though such cases occur at early gestation stages, it only became statistically significant after gd14.5.

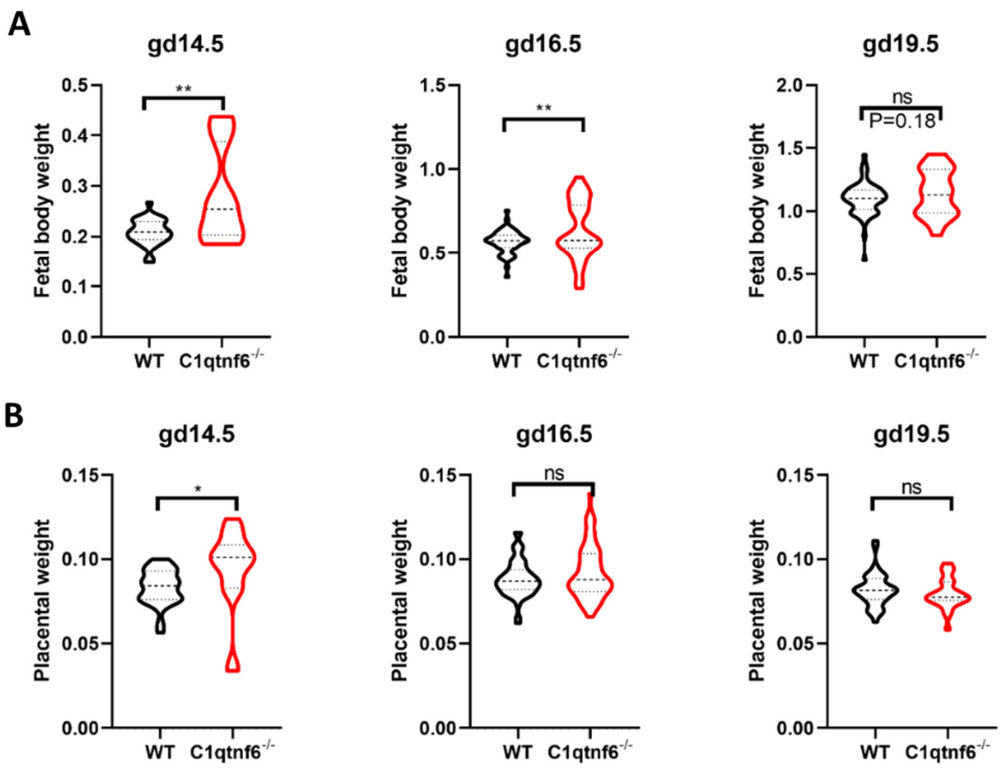

Figure S6. Comparison of the weight of retained fetuses and placentae between WT and C1qtnf6-KO at different gestation stages

This figure compares the weight of retained fetuses (A) and placentae (B) between WT and C1qtnf6-KO at different gestation stages. P-values calculated from two tailed Student's t-test are indicated.

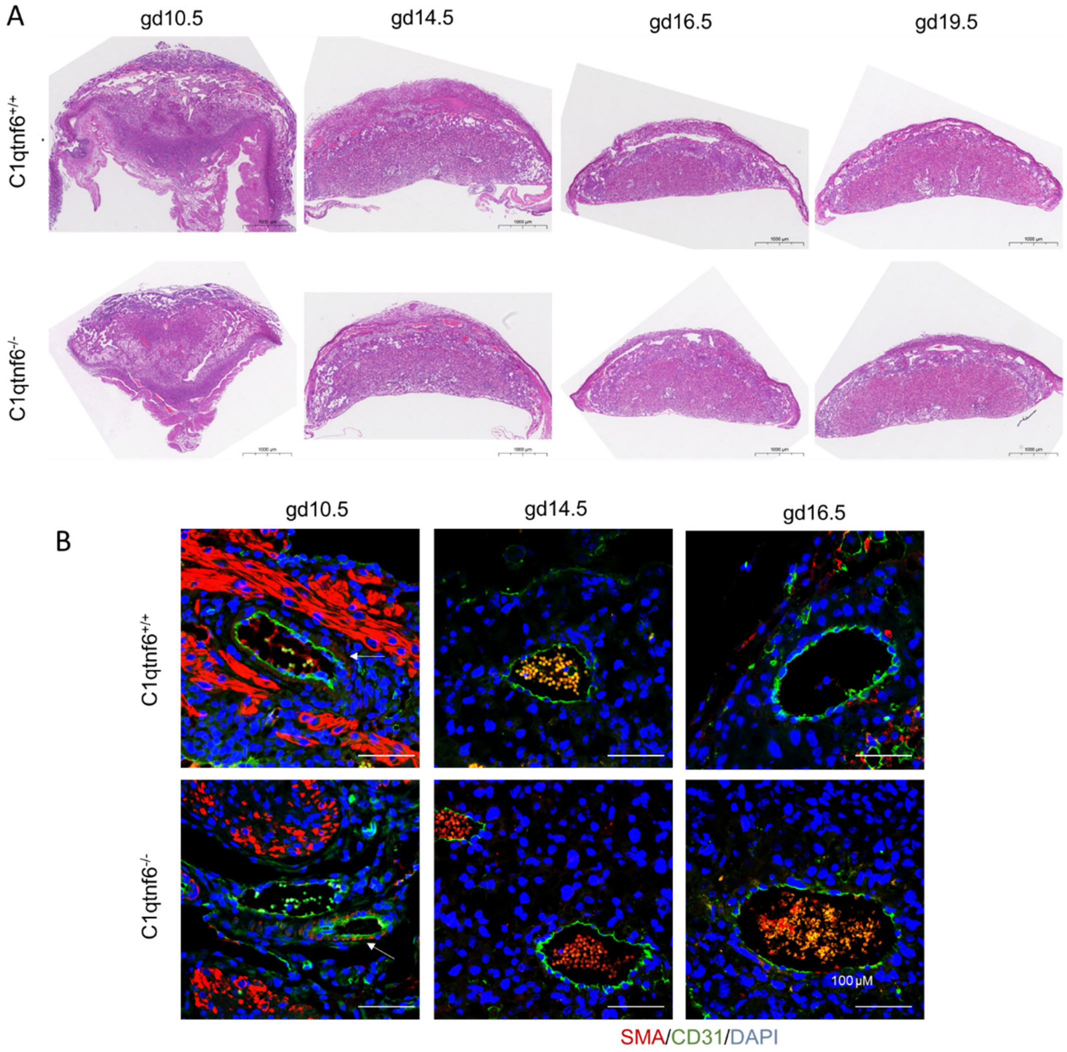

Figure S7. Placental structure and spiral artery remodeling at the MFI of WT and C1qntf6-KO mice

(A) H&E staining for the placental structure. (B) CD31/α-SMA co-immunostaining for the spiral artery remodeling in MFI.

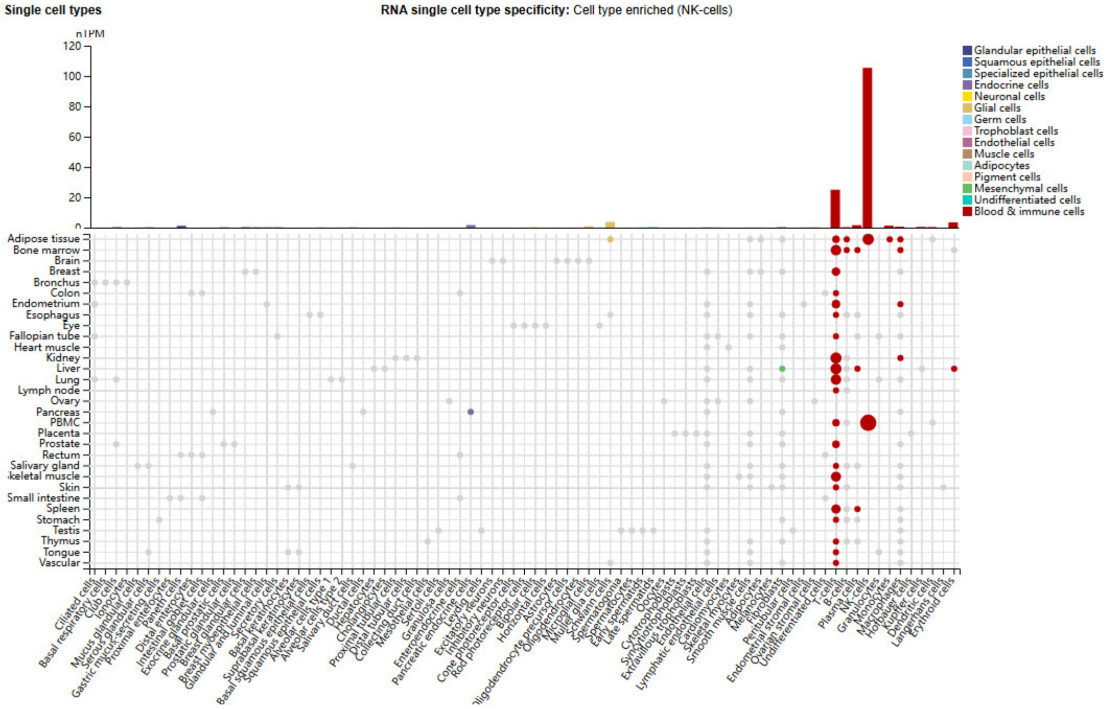

Figure S8. Enriched expression of Fcrl6 in NK cells according to HPA data

This figure shows the NK-specific expression profile of the gene Fcrl6. This figure is retrieved from HPA database.

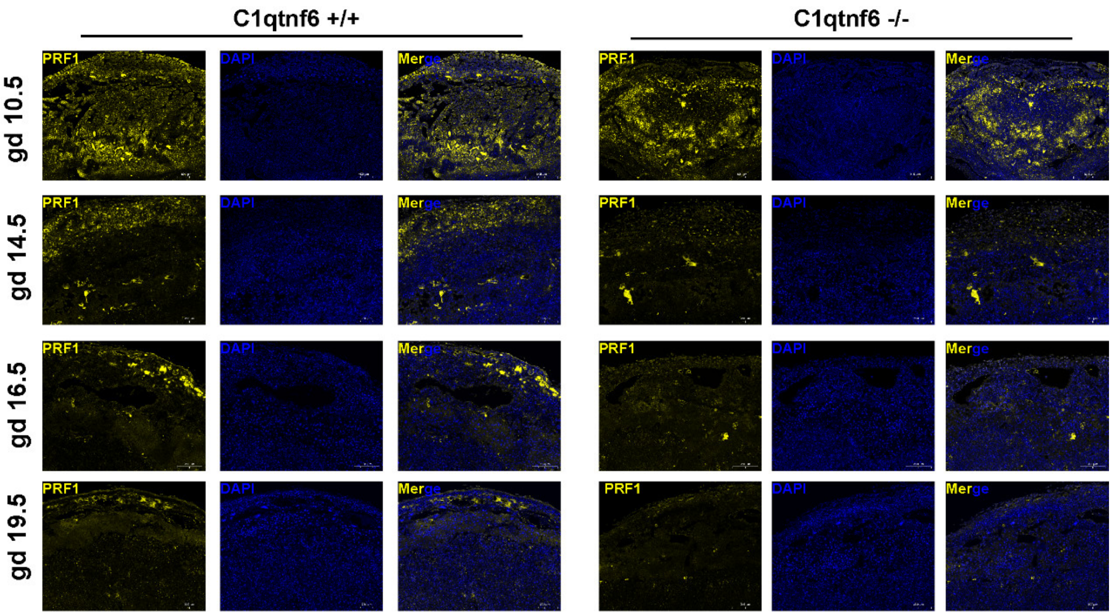

Figure S9. Immunofluorescent imaging of the PRF1 distribution at the MFI of WT and C1qtnf6-deficient mice

This figure is similar to Figure 4C, except visualized at a higher resolution of 20× zooming.

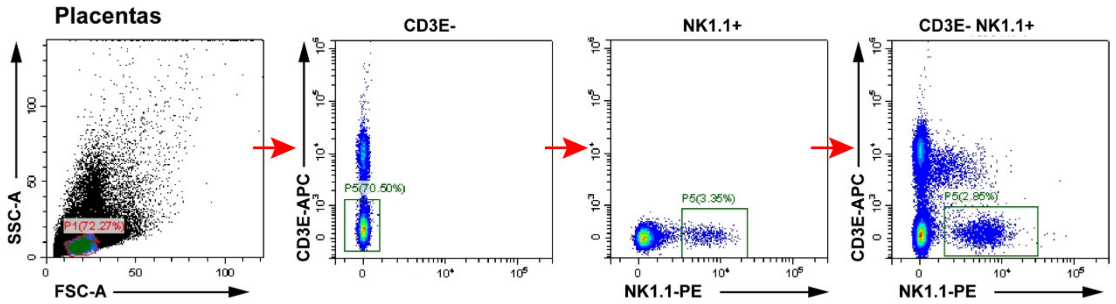

Figure S10. The gate setting for FACS analysis of dNK cells in MFI samples

This figure shows the schemes for FACS sorting of dNK cells from MFI samples. The dNK cells from MFI samples were purified by the gating strategy of live $CD3E^{-}NK1.1^{+}$.

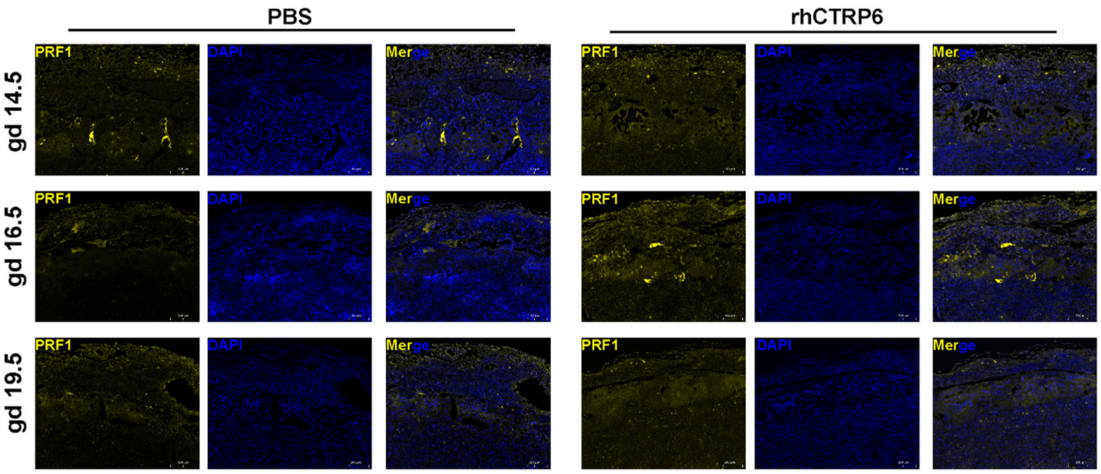

Figure S11. Immunofluorescent imaging of the PRF1 distribution at the MFI w/wo rhCTRP6 injection

This figure is similar to Figure 5C, except visualized at a higher resolution of 20× zooming.

Table S1. Sources of data used in this study, Related to Figure 1

Data type	GEO/ENA accession	Organism	Scientific name	Tissue	Placenta type	Genotype	Gestation stage	Reference
RNA-seq	GSE285241	Mouse	Mus musculus	Placenta	Hemochorial	WT	Term	This study
	GSE285241	Mouse	Mus musculus	Placenta	Hemochorial	C1qtnf6KO	Term	This study
	GSE285241	Mouse	Mus musculus	MFI	Hemochorial	WT	gd10.5	This study
	GSE285241	Mouse	Mus musculus	MFI	Hemochorial	C1qtnf6KO	gd10.5	This study
	GSE285241	Mouse	Mus musculus	MFI	Hemochorial	WT	gd14.5	This study
	GSE285241	Mouse	Mus musculus	MFI	Hemochorial	C1qtnf6KO	gd14.5	This study
	GSE285241	Mouse	Mus musculus	MFI	Hemochorial	WT	gd16.5	This study
	GSE285241	Mouse	Mus musculus	MFI	Hemochorial	C1qtnf6KO	gd16.5	This study
	GSE285241	Mouse	Mus musculus	MFI	Hemochorial	WT	gd19.5	This study
	GSE285241	Mouse	Mus musculus	MFI	Hemochorial	C1qtnf6KO	gd19.5	This study
	GSE79121	Cow	Bos taurus	Placenta	Epitheliochori	-	-	Armstrong et al., 2017, Placenta [S1]
	GSE194033	Cow	Bos taurus	Placenta	Epitheliochori	-	-	Moradi et al., 2022, BMC Genomics [S2]
	GSE79121	Dog	Canis lupus	Placenta	Endotheliocho	-	-	Armstrong et al., 2017, Placenta [S1]
	GSE79121	Armadillo	Dasypus	Placenta	Hemochorial	-	-	Armstrong et al., 2017, Placenta [S1]
	GSE46859	Horse	Equus	Placenta	Epitheliochori	-	-	Coleman et al., 2013, PLoS One [S3]
	GSE30243	Horse	Equus	Placenta	Epitheliochori	-	-	Wang et al., 2013, PNAS [S4]
RNA-seq	GSE43520	Human	Homo sapiens	Placenta	Hemochorial	-	-	Necsulea et al., 2014, Nature [S5]
	GSE118285	Human	Homo sapiens	Placenta	Hemochorial	-	-	Dunn-Fletcher et al., 2018, PLoS Biol [S6]
	GSE87708	Human	Homo sapiens	Placenta	Hemochorial	-	-	Pavličev et al., 2017, Genome Res [S7]
	GSE79121	Elephant	Loxodonta	Placenta	Endotheliocho	-	-	Armstrong et al., 2017, Placenta [S1]
	GSE153082	Rhesus	Macaca	Placenta	Hemochorial	-	-	Sun et al., 2021, Mol Biol Evol [S8]
	GSE118284	Rhesus	Macaca	Placenta	Hemochorial	-	-	Dunn-Fletcher et al., 2018, PLoS Biol [S6]
	GSE79121	Opossum	Monodelphis	Placenta	Epitheliochori	-	-	Armstrong et al., 2017, Placenta [S1]
	GSE43520	Opossum	Monodelphis	Placenta	Epitheliochori	-	-	Necsulea et al., 2014, Nature [S5]
	GSE43520	Mouse	Mus musculus	Placenta	Hemochorial	-	-	Dunn-Fletcher et al., 2018, PLoS Biol [S6]
	GSE118283	Mouse	Mus musculus	Placenta	Hemochorial	-	-	Necsulea et al., 2014, Nature [S5]
	GSE110414	Pig	Sus scrofa	Placenta	Epitheliochori	-	-	Fang et al., 2019, Int J Mol Sci [S9]
scRNA-seq	E-MTAB-	Human	Homo sapiens	MFI	Hemochorial	-	-	Vento-Tormo et al., 2018, Nature [S10]
	GSE59992	Mouse	Mus musculus	MFI	Hemochorial	-	-	Jiang et al., 2023, Cell Discov [S11]

Table S2. Litter size comparison between WT and C1qtnf6-KO mice, Related to Figure 2

	**WT-WT**	**KO-KO**
1	8	7
2	8	7
3	9	6
4	7	5
5	7	8
6	9	7
7	8	7
8	7	5
9	9	5
10	10	7
11	9	5
12	11	7
13	12	7
14	12	8
15	10	8
16	10	6
17	10	6
18	9	7
19	10	7
20	11	8
21	9	6
22	10	8
23	11	6
24	11	7
25	9	4
26	9	5
27	8	4
28	8	7
29	12	5
30	7	6
31	8	2
32	7	7
33	7	7
34	8	6
35	6	5
**total**	316	218
**Mean**	9.03	6.23
**SD**	1.62	1.36
**Mean ± SD**	9.03±1.62	6.23±1.36

Table S3. List of DEGs between MFI of WT and C1qtnf6-KO mice, Related to Figure 2

Gene ID	Gene name	log2FoldChange	padj	Stage
ENSMUSG00000031125	3830403N18Rik	1.08	0.0921766	gd16.5
ENSMUSG00000069892	9930111J21Rik2	0.81	0.0656787	gd16.5
ENSMUSG00000020473	Aebp1	-0.74	0.0240034	gd16.5
ENSMUSG00000096140	Ankrd66	0.86	0.0737773	gd16.5
ENSMUSG00000062444	Ap3b2	-1.02	0.0926150	gd16.5
ENSMUSG00000046532	Ar	-1.31	0.0227016	gd16.5
ENSMUSG00000038717	Atp5l	-0.69	0.0035497	gd19.5
ENSMUSG00000040605	Bace2	0.78	0.0628007	gd19.5
ENSMUSG00000050071	Bex1	0.74	0.0572616	gd10.5
ENSMUSG00000061132	Blnk	-0.96	0.0865014	gd16.5
ENSMUSG00000031963	Bmper	-0.68	0.0706623	gd16.5
ENSMUSG00000022440	C1qtnf6	-7.44	5.54E-55	gd10.5
ENSMUSG00000022440	C1qtnf6	-8.18	1.05E-59	gd14.5
ENSMUSG00000022440	C1qtnf6	-6.41	2.85E-40	gd16.5
ENSMUSG00000022440	C1qtnf6	-6.99	7.46E-44	gd19.5
ENSMUSG00000038521	C1s1	1.05	0.0035497	gd19.5
ENSMUSG00000004113	Cacna1b	-1.32	0.0011757	gd16.5
ENSMUSG00000057914	Cacnb2	-1.07	0.0355203	gd16.5
ENSMUSG00000039145	Camk1d	0.69	0.0628007	gd19.5
ENSMUSG00000028195	Ccn1	-0.63	0.0572616	gd10.5
ENSMUSG00000000184	Ccnd2	0.68	0.0100756	gd19.5
ENSMUSG00000046186	Cd109	-0.60	0.0020856	gd16.5
ENSMUSG00000030366	Ceacam12	2.93	0.0822818	gd10.5
ENSMUSG00000030472	Ceacam18	-1.56	2.14E-05	gd16.5
ENSMUSG00000019971	Cep290	0.70	0.0824961	gd16.5
ENSMUSG00000053063	Clec12a	0.89	0.0921719	gd16.5
ENSMUSG00000022025	Cnmd	-1.47	0.0130296	gd16.5
ENSMUSG00000032332	Col12a1	-0.78	0.0077451	gd16.5
ENSMUSG00000058806	Col13a1	-0.80	0.0710363	gd16.5
ENSMUSG00000037852	Cpe	-0.72	0.0603774	gd16.5
ENSMUSG00000025586	Cpeb1	-0.66	0.0282626	gd16.5
ENSMUSG00000024008	Cpne5	-1.70	0.0076263	gd16.5
ENSMUSG00000050967	Creg2	2.34	0.0667499	gd16.5
ENSMUSG00000023272	Creld2	-0.65	0.0474515	gd14.5
ENSMUSG00000028859	Csf3r	1.33	0.0049037	gd16.5
ENSMUSG00000040314	Ctsg	-1.53	0.0499818	gd16.5
ENSMUSG00000045382	Cxcr4	0.79	0.0500130	gd16.5
ENSMUSG00000023963	Cyp39a1	0.87	0.0130296	gd16.5
ENSMUSG00000029727	Cyp3a13	3.73	0.0921419	gd16.5
ENSMUSG00000079038	D130040H23Rik	0.71	0.0495061	gd16.5
ENSMUSG00000002297	Dbf4	0.61	0.0762988	gd16.5
ENSMUSG00000027068	Dhrs9	0.66	0.0063816	gd16.5
ENSMUSG00000018983	E2f2	-1.10	0.0181083	gd16.5
ENSMUSG00000053552	Ebf4	0.80	0.0794904	gd16.5
ENSMUSG00000050592	Fam78a	-1.29	0.0310184	gd16.5
ENSMUSG00000035509	Fbxl21	0.73	0.0821389	gd16.5
ENSMUSG00000070504	Fcrl6	-1.61	0.0082069	gd16.5
ENSMUSG00000039899	Fgl2	-1.09	0.0227016	gd16.5
ENSMUSG00000038296	Galnt18	-0.82	0.0621370	gd16.5
ENSMUSG00000094786	Gm14403	-1.37	0.0031494	gd19.5
ENSMUSG00000078867	Gm14418	-1.29	0.0055142	gd19.5

ENSMUSG00000015437	Gzmb	-1.52	0.0130296	gd16.5
ENSMUSG00000079186	Gzmc	-1.57	0.0035493	gd16.5
ENSMUSG00000059256	Gzmd	-1.65	0.0005712	gd16.5
ENSMUSG00000022156	Gzme	-1.49	0.0070072	gd16.5
ENSMUSG00000015441	Gzmf	-1.65	0.0009162	gd16.5
ENSMUSG00000040284	Gzmg	-1.71	0.0004539	gd16.5
ENSMUSG00000073409	H2-Q6	-1.07	0.0018123	gd16.5
ENSMUSG00000066842	Hmcn1	1.23	0.0092863	gd19.5
ENSMUSG00000035273	Hpse	-0.71	0.0865912	gd16.5
ENSMUSG00000070407	Hs3st3b1	0.70	0.0328846	gd19.5
ENSMUSG00000027871	Hsd3b1	4.14	0.0294417	gd16.5
ENSMUSG00000070687	Htr1d	1.22	0.0667499	gd16.5
ENSMUSG00000032394	Igdcc3	-1.25	0.0586400	gd16.5
ENSMUSG00000070427	Il18bp	0.65	0.0667499	gd16.5
ENSMUSG00000003477	Inmt	-1.59	0.0656787	gd16.5
ENSMUSG00000079553	Kifc1	-0.77	0.0294417	gd16.5
ENSMUSG00000032036	Kirrel3	-1.20	0.0157733	gd16.5
ENSMUSG00000055193	Klk15	-0.92	0.0704857	gd16.5
ENSMUSG00000006948	Klk4	-0.95	0.0130296	gd16.5
ENSMUSG00000038668	Lpar1	-0.74	0.0844766	gd16.5
ENSMUSG00000027377	Mall	-0.63	0.0657028	gd16.5
ENSMUSG00000022324	Matn2	-0.76	0.0107538	gd16.5
ENSMUSG00000022157	Mcpt8	-2.23	0.0997883	gd16.5
ENSMUSG00000027239	Mdk	0.99	0.0107750	gd19.5
ENSMUSG00000035299	Mid1	1.93	0.0313376	gd14.5
ENSMUSG00000038022	Mindy4	-0.80	0.0794904	gd16.5
ENSMUSG00000050276	Mrgprg	-1.02	0.0656787	gd16.5
ENSMUSG00000102758	Naaladl2	-1.30	0.0667287	gd16.5
ENSMUSG00000078942	Naip6	1.72	0.0486823	gd16.5
ENSMUSG00000015950	Ncf1	0.91	0.0407273	gd16.5
ENSMUSG00000033585	Ndn	-0.60	0.0417290	gd16.5
ENSMUSG00000035121	Neil2	0.86	0.0181132	gd16.5
ENSMUSG00000004891	Nes	0.59	0.0931667	gd19.5
ENSMUSG00000000120	Ngfr	-2.29	0.0086057	gd16.5
ENSMUSG00000021806	Nid2	-0.74	0.0518074	gd16.5
ENSMUSG00000029019	Nppb	1.96	0.0011757	gd16.5
ENSMUSG00000066861	Oas1g	0.75	0.0070072	gd16.5
ENSMUSG00000067780	Pi15	-2.13	0.0500130	gd16.5
ENSMUSG00000058818	Pirb	1.16	0.0495061	gd16.5
ENSMUSG00000054580	Pla2r1	-0.90	0.0478702	gd16.5
ENSMUSG00000016495	Plgrkt	1.32	0.0540001	gd16.5
ENSMUSG00000039457	Ppl	-0.67	0.0794904	gd16.5
ENSMUSG00000046442	Ppm1e	-0.71	0.0880683	gd16.5
ENSMUSG00000037202	Prf1	-1.83	0.0005712	gd16.5
ENSMUSG00000038883	Prl3a1	0.91	0.0256223	gd16.5
ENSMUSG00000021347	Prl7b1	0.74	0.0770802	gd16.5
ENSMUSG00000060738	Prl7c1	0.96	0.0256223	gd16.5
ENSMUSG00000021345	Prl8a6	1.40	0.0822818	gd10.5
ENSMUSG00000003526	Prodh	0.62	0.0770802	gd16.5
ENSMUSG00000027347	Rasgrp1	-0.89	0.0355203	gd16.5
ENSMUSG00000042129	Rassf4	0.64	0.0865912	gd16.5
ENSMUSG00000074457	S100a16	-0.61	0.0150534	gd16.5
ENSMUSG00000056071	S100a9	1.07	0.0625577	gd16.5
ENSMUSG00000067586	S1pr3	0.71	0.0128299	gd19.5

ENSMUSG00000040026	Saa3	4.08	0.0011757	gd16.5
ENSMUSG00000085272	Sbk3	-0.96	0.0741861	gd16.5
ENSMUSG00000032018	Sc5d	0.64	0.0478702	gd16.5
ENSMUSG00000075316	Scn9a	1.04	0.0070072	gd16.5
ENSMUSG00000021904	Sema3g	-0.86	0.0183376	gd16.5
ENSMUSG00000023224	Serping1	-1.01	0.0070072	gd16.5
ENSMUSG00000073494	Sh2d1b2	-1.75	0.0070072	gd16.5
ENSMUSG00000030108	Slc6a13	-1.34	0.0657028	gd16.5
ENSMUSG00000063632	Sox11	1.21	0.0676478	gd16.5
ENSMUSG00000046470	Sox18	-0.62	0.0818203	gd16.5
ENSMUSG00000080316	Spaca6	0.76	0.0070072	gd16.5
ENSMUSG00000038156	Spon1	-1.20	0.0107538	gd16.5
ENSMUSG00000063529	Stmnd1	1.93	0.0737773	gd16.5
ENSMUSG00000029272	Sult1e1	1.20	0.0019436	gd16.5
ENSMUSG00000039231	Suv39h1	-0.71	0.0534406	gd16.5
ENSMUSG00000026646	Suv39h2	1.01	0.0657716	gd16.5
ENSMUSG00000068923	Syt11	0.79	0.0092388	gd19.5
ENSMUSG00000054003	Tdrd9	-1.57	0.0931735	gd16.5
ENSMUSG00000035493	Tgfbi	-0.74	0.0074765	gd16.5
ENSMUSG00000037731	Themis2	1.02	0.0898905	gd16.5
ENSMUSG00000032289	Thsd4	-0.68	0.0976030	gd16.5
ENSMUSG00000032011	Thy1	-1.25	0.0997883	gd16.5
ENSMUSG00000062545	Tlr12	-0.95	0.0419890	gd16.5
ENSMUSG00000024245	Tmem178	1.49	0.0107750	gd19.5
ENSMUSG00000040046	Tph1	3.12	0.0625577	gd16.5
ENSMUSG00000021573	Tppp	1.19	0.0480971	gd16.5
ENSMUSG00000050747	Trim15	1.41	0.0710363	gd16.5
ENSMUSG00000052749	Trim30b	1.44	0.0931735	gd16.5
ENSMUSG00000062591	Tubb4a	-0.83	0.0256223	gd16.5
ENSMUSG00000001403	Ube2c	-0.64	0.0710363	gd16.5
ENSMUSG00000012126	Ubxn11	-1.19	0.0931667	gd19.5
ENSMUSG00000057948	Unc13d	-0.61	0.0790893	gd16.5
ENSMUSG00000068457	Uty	20.52	1.62E-09	gd10.5
ENSMUSG00000021614	Vcan	1.06	0.0917211	gd16.5
ENSMUSG00000031380	Vegfd	-0.91	0.0455359	gd16.5
ENSMUSG00000017723	Wfdc2	-1.55	0.0415134	gd16.5
ENSMUSG00000051951	Xkr4	-1.32	0.0355203	gd16.5
ENSMUSG00000018427	Ypel2	0.59	0.0672970	gd16.5
ENSMUSG00000045333	Zfp423	-0.92	0.0043097	gd16.5
ENSMUSG00000078866	Zfp970	-1.36	4.82E-05	gd19.5
ENSMUSG00000021327	Zkscan3	-0.75	0.0003555	gd19.5

Table S4. gRNA target sequence, Related to STAR methods

Primer name	Sequence
gRNA1	CAATGCTCGACCATGTTGCTGGG
gRNA2	AATTCTCTGGGAGGCACGATGGG
gRNA3	TTGGTCCAGCATGAATGGTAGGG
gRNA4	TTTCCAGGCTAGGTCAGTAAAGG

Table S5. The genotyping PCR primers, Related to STAR methods

Primer name	Sequence	Size (bp)
Primer 1-F	5'-CAAGGGTGAGGGACATTTTACAGG-3'	650
Primer 1-R	5'-GATTTCCAAGCAGGTACAAAAGGTA-3'	
Primer 1-F	5'-CAAGGGTGAGGGACATTTTACAGG-3'	574
Primer 2-R	5'-GAGTCAAATGCCCTGGGAGTTA-3'	

Note: Homozygotes: one band with 650 bp.

Heterozygotes: two bands with 650 bp and 574 bp.

Wildtype allele: one band with 574 bp.

Table S6. The real-time PCR primer sets, Related to STAR methods

Primer	Targeted	Sequences (5’-3’)
C1qtnf6-F	*C1qtnf6*	5’-CAGGGAAGCCAGGGTCTTTG-3’
C1qtnf6-R		5’-ACGATGTGCACGTAGGTCTC-3’
C1qtnf1-F	*C1qtnf1*	5’-CCTTGTGCCACGAGTTCAGG-3’
C1qtnf1-R		5’-CGCCTTTCAGGATGGTGATG-3’
C1qtnf2-F	*C1qtnf2*	5’-ACTCTTGGCCTGTGCCCTTC-3’
C1qtnf2-R		5’-CCCATTCTTCCCACCACTCC-3’
C1qtnf3-F	*C1qtnf3*	5’-CTTCAGCATGTACAGCTATG-3’
C1qtnf3-R		5’-GTTGCCCATTCTTAGCCAGACT-3’
C1qtnf4-F	*C1qtnf4*	5’-GACGCTGTCGGTGAAGCTGA-3’
C1qtnf4-R		5’-CTTGCCGTGGTTGCTGTAGG-3’
C1qtnf5-F	*C1qtnf5*	5’-GGGCCATTACGACCCCACTA-3’
C1qtnf5-R		5’-GTAATCACCCACGCCCACCT-3’
C1qtnf7-F	*C1qtnf7*	5’-CTCGGGCCAATCAGGCTAAG-3’
C1qtnf7-R		5’-GTGCCCTTTTCCCCCTTCTC-3’
C1qtnf8-F	*C1qtnf8*	5’-ACGGCCCACTATAGACATCGAA-3’
C1qtnf8-R		5’-TGTAGTTCCAGGTGTGCACGTT-3’
C1qtnf9-F	*C1qtnf9*	5’-AGGGGCAGAAGGGGGATAAA-3’
C1qtnf9-R		5’-CGCCTTTCCAGCCTCTCATT-3’
C1qtnf10-F	*C1qtnf10*	5’-CGAAGGCTACGAGGTGCTCA-3’
C1qtnf10-R		5’-GCTGTTGCTGGCGTAGTCGT-3’
C1qtnf11-F	*C1qtnf11*	5’-TGAAGTGTTGCGCTTTGACG-3’
C1qtnf11-R		5’-GATCCGCATCCTGAGCAATG-3’
C1qtnf12-F	*C1qtnf12*	5’-GCTGGTGGTTGAGGCCTTCT-3’
C1qtnf12-R		5’-GCAGAGACTGGGGCTGTGAA-3’
C1qtnf13-F	*C1qtnf13*	5’-GTCCCTGCCCACTTTCATCC-3’
C1qtnf13-R		5’-GCCAGCGTAAAAGGCGATCT-3’
C1qtnf14-F	*C1qtnf14*	5’-CAGCACGGCCACCTATACCA-3’
C1qtnf14-R		5’-TTGCAGAGGTCTGCCCACAT-3’
C1qtnf15-F	*C1qtnf15*	5’-CGGACCTGTCCCCAAATCAG-3’
C1qtnf15-R		5’-AGGGTGGGGTGTTTCCACCT-3’
Prf1-F	*Prf1*	5’-ACGCATGATCTGCTCTTCG-3’
Prf1-R		5’-GTCCTGGTTGGTGACCTTTG-3’
Gzmb-F	*Gzmb*	5’-GTGCGGGGGACCCAAAGACCAAAC-3’
Gzmb-R		5’-GCACGTGGAGGTGAACCATCCTTATAT-3’
Gzmc-F	*Gzmc*	5’-TGACCCTACTTCTGCC-3’
Gzmc-R		5’-CTCCTCCTTAGCCTTG-3’
Gzmd-F	*Gzmd*	5’-TTCTCCTGACCCTACTTCTG-3’
Gzmd-R		5’-CTCCTCCTTAGCCGTGAT-3’
Gzme-F	*Gzme*	5’-GATTCTCCTGACCCTACTTC-3’
Gzme-R		5’-CTCCTCCTTAGCCTTGAT-3’
Gapdh-F	*Gapdh*	5’-GGAGAGTGTTTCCTCGTCCC-3’
Gapdh-R		5’-ACTGTGCCGTTGAATTTGCC-3’

Table S7. Antibody used in this study, Related to STAR methods

Antibody	Manufacture (catalogue number)	Source	Applications (working dilution)	Website Link
**CTRP6**	Abcam (ab36900)	Rabbit	IHC (1:200)<br>WB (1:1000)	https://www.abcam.com/en-us/products/primary-antibodies/ctrp6-antibody-ab36900
**Prf1**	Santa Cruz (sc-374346)	Mouse	IHC (1:50)<br>WB (1:1000)	https://www.scbt.com/p/perforin-1-antibody-e-5
**Gzmb**	R&D systems (Af1865-SE)	Mouse	WB (0.1ug/mL)	https://www.rndsystems.com/cn/products/mouse-granzyme-b-antibody_af1865
**C3**	Abcam (ab181147)	Rabbit	WB (1:1000)	https://www.abcam.cn/products/primary-antibodies/c3-antibody-epr2988-ab181147
**GAPDH**	Proteintech (10494-1-AP)	Mouse	WB (1:50000)	https://www.ptglab.co.jp/products/GAPDH-Antibody-10494-1-AP.htm
**HSP90**	Proteintech (60318-1-lg)	Mouse	WB (1:50000)	https://www.ptglab.com/products/HSP90-Antibody-60318-1-Ig.htm

REFERENCES

[S1] Armstrong, D.L., McGowen, M.R., Weckle, A., Pantham, P., Caravas, J., Agnew, D., Benirschke, K., Savage-Rumbaugh, S., Nevo, E., Kim, C.J., et al. (2017). The core transcriptome of mammalian placentas and the divergence of expression with placental shape. Placenta 57, 71-78. 10.1016/j.placenta.2017.04.015.

[S2] Moradi, M., Zhandi, M., Sharafi, M., Akbari, A., Atrabi, M.J., and Totonchi, M. (2022). Gene expression profile of placentomes and clinical parameters in the cows with retained placenta. BMC Genomics 23, 760. 10.1186/s12864-022-08989-5.

[S3] Coleman, S.J., Zeng, Z., Hestand, M.S., Liu, J., and Macleod, J.N. (2013). Analysis of unannotated equine transcripts identified by mRNA sequencing. PLoS One 8, e70125. 10.1371/journal.pone.0070125.

[S4] Wang, X., Miller, D.C., Harman, R., Antczak, D.F., and Clark, A.G. (2013). Paternally expressed genes predominate in the placenta. Proc Natl Acad Sci U S A 110, 10705-10710. 10.1073/pnas.1308998110.

[S5] Necsulea, A., Soumillon, M., Warnefors, M., Liechti, A., Daish, T., Zeller, U., Baker, J.C., Grutzner, F., and Kaessmann, H. (2014). The evolution of lncRNA repertoires and expression patterns in tetrapods. Nature 505, 635-640. 10.1038/nature12943.

[S6] Dunn-Fletcher, C.E., Muglia, L.M., Pavlicev, M., Wolf, G., Sun, M.A., Hu, Y.C., Huffman, E., Tumukuntala, S., Thiele, K., Mukherjee, A., et al. (2018). Anthropoid primate-specific retroviral element THE1B controls expression of CRH in placenta and alters gestation length. PLoS Biol 16, e2006337. 10.1371/journal.pbio.2006337.

[S7] Pavlicev, M., Wagner, G.P., Chavan, A.R., Owens, K., Maziarz, J., Dunn-Fletcher, C., Kallapur, S.G., Muglia, L., and Jones, H. (2017). Single-cell transcriptomics of the human placenta: inferring the cell communication network of the maternal-fetal interface. Genome Res 27, 349-361. 10.1101/gr.207597.116.

[S8] Sun, M.A., Wolf, G., Wang, Y., Senft, A.D., Ralls, S., Jin, J., Dunn-Fletcher, C.E., Muglia, L.J., and Macfarlan, T.S. (2021). Endogenous Retroviruses Drive Lineage-Specific Regulatory Evolution across Primate and Rodent Placentae. Mol Biol Evol 38, 4992-5004. 10.1093/molbev/msab223.

[S9] Fang, X., Nevo, E., Han, L., Levanon, E.Y., Zhao, J., Avivi, A., Larkin, D., Jiang, X., Feranchuk, S., Zhu, Y., et al. (2014). Genome-wide adaptive complexes to underground stresses in blind mole rats Spalax. Nat Commun 5, 3966. 10.1038/ncomms4966.

[S10] Vento-Tormo, R., Efremova, M., Botting, R.A., Turco, M.Y., Vento-Tormo, M., Meyer, K.B., Park, J.E., Stephenson, E., Polanski, K., Goncalves, A., et al. (2018). Single-cell reconstruction of the early maternal-fetal interface in humans. Nature 563, 347-353. 10.1038/s41586-018-0698-6.

[S11] Jiang, X., Wang, Y., Xiao, Z., Yan, L., Guo, S., Wang, Y., Wu, H., Zhao, X., Lu, X., and Wang, H. (2023). A differentiation roadmap of murine placentation at single-cell resolution. Cell Discov 9, 30. 10.1038/s41421-022-00513-z.
